# DNA Repair Pathway Selection Caused by Defects in *TEL1*, *SAE2*, and *De Novo* Telomere Addition Generates Specific Chromosomal Rearrangement Signatures

**DOI:** 10.1371/journal.pgen.1004277

**Published:** 2014-04-03

**Authors:** Christopher D. Putnam, Katielee Pallis, Tikvah K. Hayes, Richard D. Kolodner

**Affiliations:** 1 Ludwig Institute for Cancer Research, University of California School of Medicine, San Diego, La Jolla, California, United States of America; 2 Department of Medicine, University of California School of Medicine, San Diego, La Jolla, California, United States of America; 3 Department of Cellular and Molecular Medicine, University of California School of Medicine, San Diego, La Jolla, California, United States of America; 4 Moores-UCSD Cancer Center, University of California School of Medicine, San Diego, La Jolla, California, United States of America; 5 Institute of Genomic Medicine, University of California School of Medicine, San Diego, La Jolla, California, United States of America; Stanford University School of Medicine, United States of America

## Abstract

Whole genome sequencing of cancer genomes has revealed a diversity of recurrent gross chromosomal rearrangements (GCRs) that are likely signatures of specific defects in DNA damage response pathways. However, inferring the underlying defects has been difficult due to insufficient information relating defects in DNA metabolism to GCR signatures. By analyzing over 95 mutant strains of *Saccharomyces cerevisiae*, we found that the frequency of GCRs that deleted an internal *CAN1/URA3* cassette on chrV L while retaining a chrV L telomeric *hph* marker was significantly higher in *tel1Δ*, *sae2Δ*, *rad53Δ sml1Δ*, and *mrc1Δ tof1Δ* mutants. The *hph*-retaining GCRs isolated from *tel1Δ* mutants contained either an interstitial deletion dependent on non-homologous end-joining or an inverted duplication that appeared to be initiated from a double strand break (DSB) on chrV L followed by hairpin formation, copying of chrV L from the DSB toward the centromere, and homologous recombination to capture the *hph*-containing end of chrV L. In contrast, *hph*-containing GCRs from other mutants were primarily interstitial deletions (*mrc1Δ tof1Δ*) or inverted duplications (*sae2Δ* and *rad53Δ sml1Δ*). Mutants with impaired *de novo* telomere addition had increased frequencies of *hph*-containing GCRs, whereas mutants with increased *de novo* telomere addition had decreased frequencies of *hph*-containing GCRs. Both types of *hph*-retaining GCRs occurred in wild-type strains, suggesting that the increased frequencies of *hph* retention were due to the relative efficiencies of competing DNA repair pathways. Interestingly, the inverted duplications observed here resemble common GCRs in metastatic pancreatic cancer.

## Author Summary

Recent advances in the sequencing of human cancer genomes have revealed that some types of genome rearrangements are more common in specific types of cancers. Thus, these cancers may share defects in DNA repair mechanisms, which may play roles in initiation or progression of the disease and may be useful therapeutically. Linking a common rearrangement signature to a specific genetic or epigenetic alteration is currently challenging, because we do not know which rearrangement signatures are linked to which DNA repair defects. Here we used a genetic assay in the model organism *Saccharomyces cerevisiae* to specifically link two classes of chromosomal rearrangements, interstitial deletions and inverted duplications, to specific genetic defects. These results begin to map out the links between observed chromosomal rearrangements and specific DNA repair defects and in the present case, may provide insights into the chromosomal rearrangements frequently observed in metastatic pancreatic cancer.

## Introduction

Large numbers of complex chromosomal rearrangements (called gross chromosomal rearrangements or GCRs) are seen in many cancers, potentially due to ongoing genome instability. Much of our present knowledge on the genome rearrangements seen in cancer is from cytogenetic observations of large-scale genome rearrangements and processes associated with their formation. Some examples include cytogenetically observable genome rearrangements that appear to be triggered by dicentric chromosomes undergoing cycles of bridge-fusion-breakage [1]–[3] or breakage of chromosomes by anaphase bridges that have been observed in early stages of carcinogenesis [4] and in cells containing defects in cancer susceptibility genes like *BLM* [5]. The advent of genomics methods including whole-genome next generation sequencing of the genomes from tumors and paired normal tissue has greatly expanded the information available about the kinds of somatic GCRs present in cancers. Interestingly, some types of GCRs may be specifically enhanced in subsets of cancer, including retrotransposition events in colorectal cancers [6], inversions in pancreatic cancer [7], tandem duplications in ovarian and triple-negative breast cancer [8], [9], and focal copy number changes in ovarian cancer [10]. The presence of these rearrangements in a subset of cancers of a specific type suggests that the genetic background in different cancers may influence the mechanisms of GCRs formation. The limited understanding of the types of genetic defects that affect GCR formation and the enormous genetic variation seen in many cancers pose challenges to understanding the influence of genetic background on the types of GCRs seen and their rates of formation.

Quantitative measurements of the accumulation of GCRs in the yeast *Saccharomyces cerevisiae* have been useful for identifying pathways that normally suppress the formation of GCRs. These measurements have typically measured the loss of genetic markers present on a non-essential terminal region of chromosomes in haploid strains [11]–[15]. This feature of these assays allows the formation of different types of GCRs by a diversity of mechanisms depending on the assay and the genotype of the strain used. The types of GCRs observed include terminal deletions healed by *de novo* telomere addition, simple monocentric translocations including the formation of circular chromosomes, and complex GCRs that are initiated by the formation of dicentric translocations and end-to-end chromosome fusions followed by multi-step rearrangements that resolve the initial dicentric translocations to monocentric GCRs [11], [14], [16]–[20].

During the analysis of GCRs formed in assays utilizing a *CAN1/URA3* cassette placed at various locations along the left arm of chromosome V (chrV L; [14], [21]), we noticed that a high proportion of GCRs in some mutants, including *tel1Δ*, *sae2Δ*, *mrc1Δ tof1Δ* and *rad53Δ sml1Δ*, retained a hygromycin resistance marker (*hph*) present on the assay chromosome telomeric to the *CAN1/URA3* cassette. We initially characterized the GCRs formed in the *tel1Δ* mutant, which lacks the gene encoding a DNA damage checkpoint protein kinase that is important for telomere maintenance [16], [22]–[25], and determined that *hph*-retention was due to the formation of interstitial deletions by non-homologous end-joining (NHEJ) or by formation of inverted duplications that were then resolved by homologous recombination (HR) between the *ura3-52* allele (a Ty element insertion at the *URA3* locus in the duplicated region) and *URA3* in the *CAN1/URA3* cassette. Both types of *hph*+ products were observed in wild-type strains, but at much lower frequencies than in the *tel1Δ* mutant. Importantly, the *hph*− GCRs formed in the *tel1Δ* mutant were also associated with increased frequencies of inverted duplications that differed from the *hph*+ GCRs only with respect to the homologies used for telomere capture. Deletion of *SAE2* also caused an increase in *hph* retention. However, unlike the *tel1Δ* mutation, this increase was solely due to increased levels of inverted duplications. Detailed analysis of the interactions between *tel1* and *sae2* single mutations and mutations affecting the Mre11-Rad50-Xrs2 (MRX) complex, which functions in the resection of DNA at double-stranded breaks (DSBs), or the DNA damage checkpoint revealed that complex interactions between repair pathways promote the formation of specific rearrangements. Furthermore, genetic defects that suppressed *de novo* telomere addition increased *hph* retention, whereas genetic defects that increased *de novo* telomere addition decreased *hph* retention. Together, these results suggest a mechanism by which Tel1, Sae2, and *de novo* telomere addition play a role in suppressing inverted duplications and, in some cases, interstitial deletions, and further demonstrate that defects in these pathways/genes result in GCRs with a specific structural signature.

## Results

### Retention of telomeric DNA in GCRs is assay- and genotype-specific

**Figure 1:**
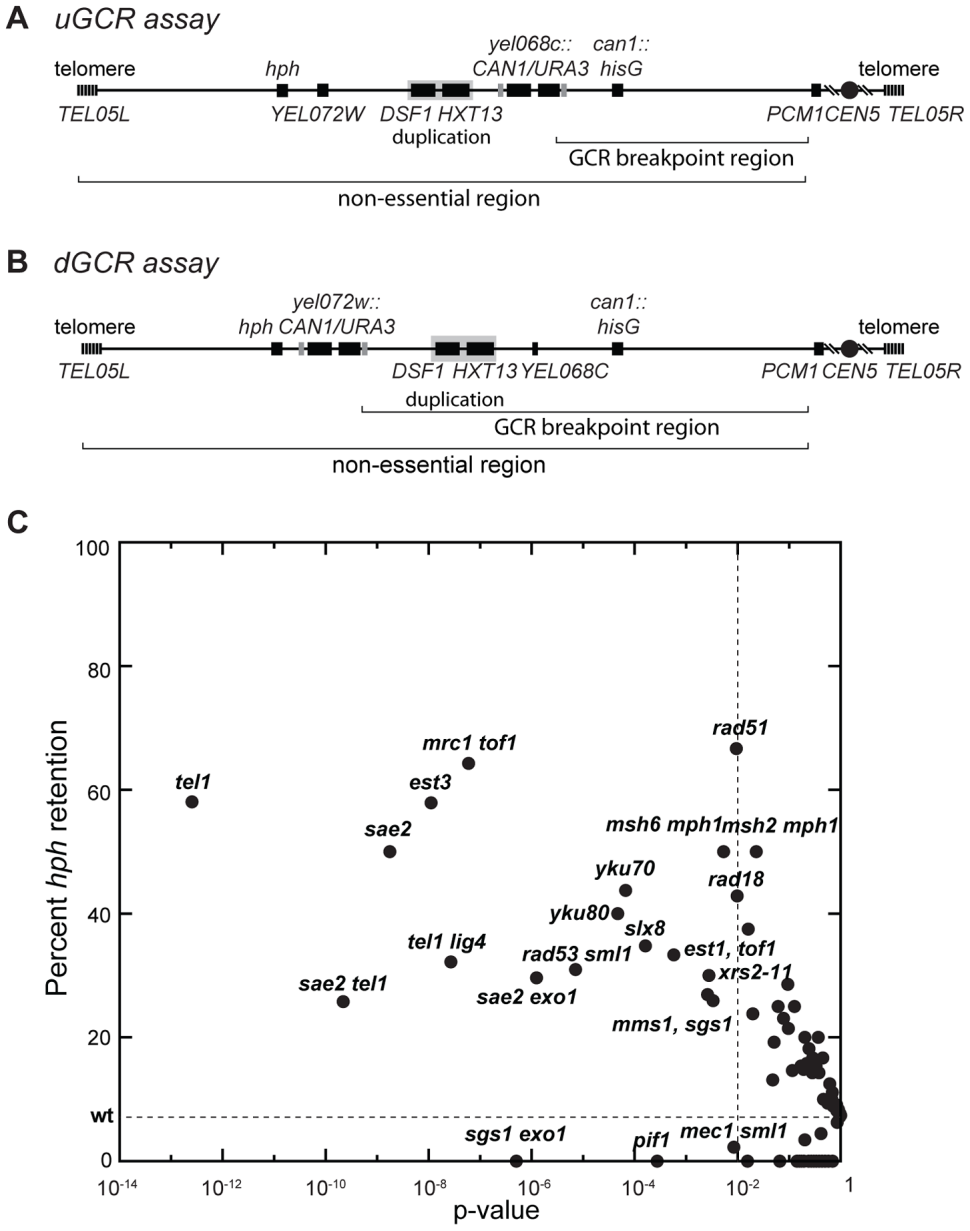
Biased distribution of GCRs retaining *hph*. (**A and B**) Schematic showing the positions of the *CAN1/URA3* cassette in the uGCR and dGCR assays relative to the 4.2 kb *HXT13-DSF1* segmental duplication on chrV. The GCR breakpoint region (horizontal bracket) is the region in which rearrangements must occur to lose *CAN1/URA3* cassette but not the essential gene *PCM1*. (**C**) Plot of the percent retention of *hph* in the uGCR assay in various mutant backgrounds against the respective p-value for retention (G-test) using the wild-type distribution (2 of 27) as the expected distribution. These data include strains generated and analyzed in this study. Points to the left of the vertical dashed line correspond to mutations with p-values<0.01. The horizontal dashed line is the frequency of *hph* retention in the wild-type uGCR assay strain.

Two GCR assays on chrV that incorporated a telomeric hygromycin resistance marker (*hph*) (Figure 1A and B) [14] were used to characterize the GCR rate and the frequency of GCRs retaining *hph* in over 95 mutant strains [14], [21]. The *yel072w::CAN1/URA3* GCR (dGCR) assay primarily mediates GCRs by duplication-mediated rearrangements with chromosomes IV, X, and XIV; the GCRs derived using this assay frequently lost the telomeric portion of chrV that includes the *hph* marker [14], [21]. Consistent with this, 0 of 62 GCRs (0%) derived in the wild-type dGCR assay strain and 15 of 2435 GCRs (0.6%) formed in all tested dGCR assay strains retained *hph*. In contrast, the frequency of *hph* retention was higher in the GCRs formed in the *yel068c::CAN1/URA3* GCR (uGCR) assay, which mediates GCRs by single copy or “unique” genomic sequences. In the wild-type uGCR assay strain, 2 of the 27 GCR-containing isolates (7%) retained *hph*, and 367 of 2670 GCRs (14%) formed in all tested uGCR assay strains retained the *hph* marker. Specific mutations significantly increased the frequency of *hph+* GCRs relative to wild type (Figure 1C). These mutations included *tel1Δ* (58% *hph+*; p = 3×10^−13^, G-test), *sae2Δ* (50% *hph*+; p = 2×10^−9^, G-test), *rad53Δ sml1Δ* (31% *hph+*; p = 7×10^−6^, G-test), and *mrc1Δ tof1Δ* (64% *hph*+; p = 6×10^−8^, G-test).

### *hph+* uGCRs from the *tel1Δ* strain are either interstitial deletions or inverted duplications

**Figure 2:**
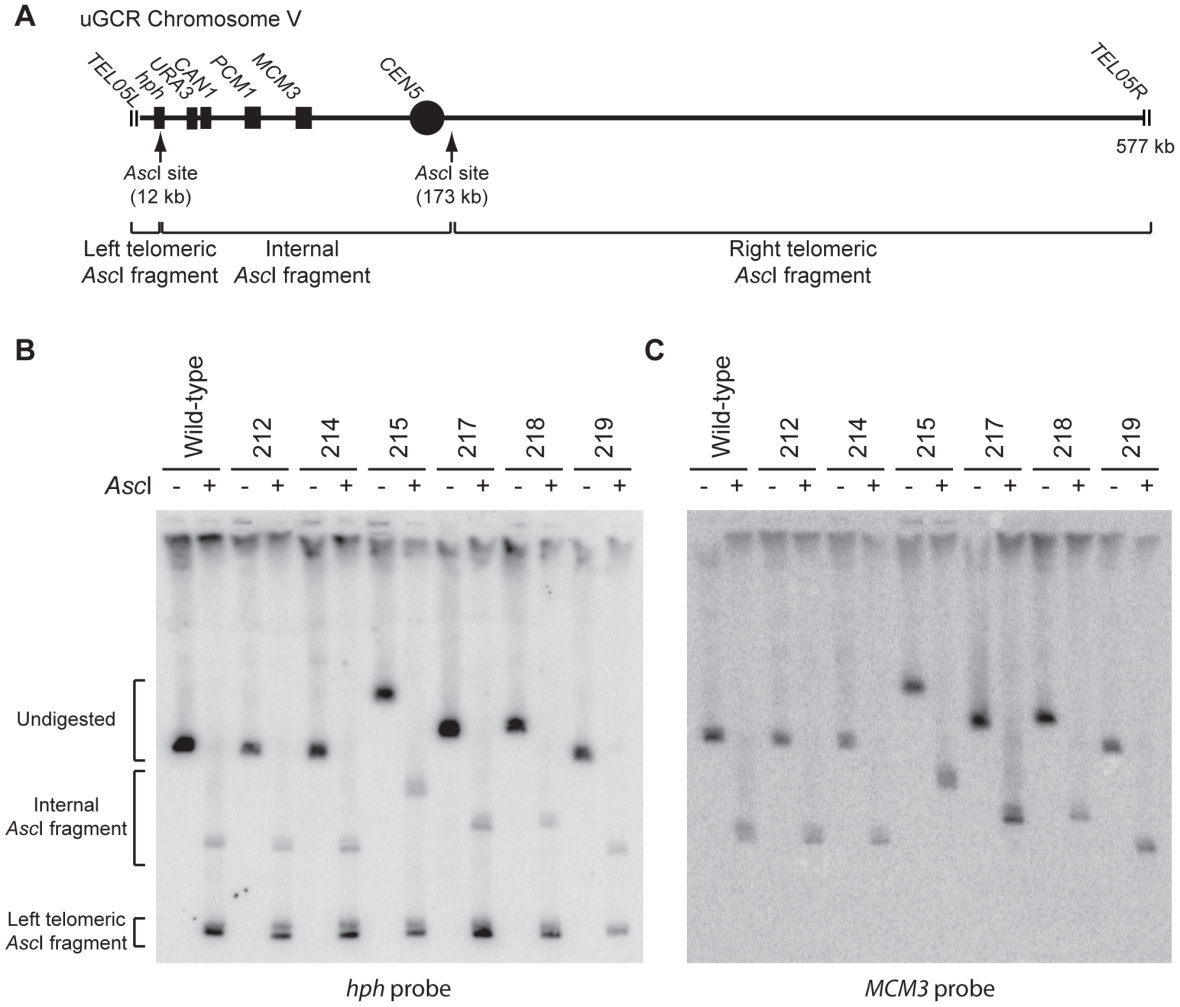
GCRs retaining *hph* belong to two size classes. (**A**) Digestion of the uGCR chrV divides the uGCR chrV into left telomeric, internal, and right telomeric fragments. Vertical arrows indicate the *Asc*I cleavage sites and relevant chromosomal features are labeled. (**B**) Southern blot using an *hph* probe of a pulsed-field gel (PFG) with DNA from the wild-type strain (RDKY6677) and 6 GCR-containing isolates (212, 214, 215, 217, 218, and 219) with and without *Asc*I digestion. The *hph* probe hybridizes to the intact chromosome and the internal and left telomeric fragments. (**C**) Southern blot of a second PFG with the same samples as in panel B using an *MCM3* probe. The *MCM3* probe hybridizes to the intact chromosome and the internal fragment.

We characterized 18 *hph*+ GCRs isolated from the *tel1Δ* uGCR assay strain by pulsed-field gel electrophoresis (PFGE) and Southern blotting. Probes complementary to *hph* and to *MCM3*, which is an essential gene on chrV, hybridized to the same band in lanes with undigested chromosomes (Figures 2 and S1), indicating that *hph* was retained on chrV. The size of chrV was similar to wild-type in 8 isolates and was larger than wild-type in 10 isolates. Digestion of chrV by *Asc*I generates three fragments from the starting chromosome: the left telomeric fragment contains *hph* sequence, the internal fragment contains both *hph* and *MCM3* sequence, and the right telomeric fragment has neither *hph* nor *MCM3* sequence (Figure 2A). In all cases with a larger than wild-type chrV, the change in size appeared to be due to changes in the internal *Asc*I fragment (Figure 2B and C; Figure S1).

**Figure 3:**
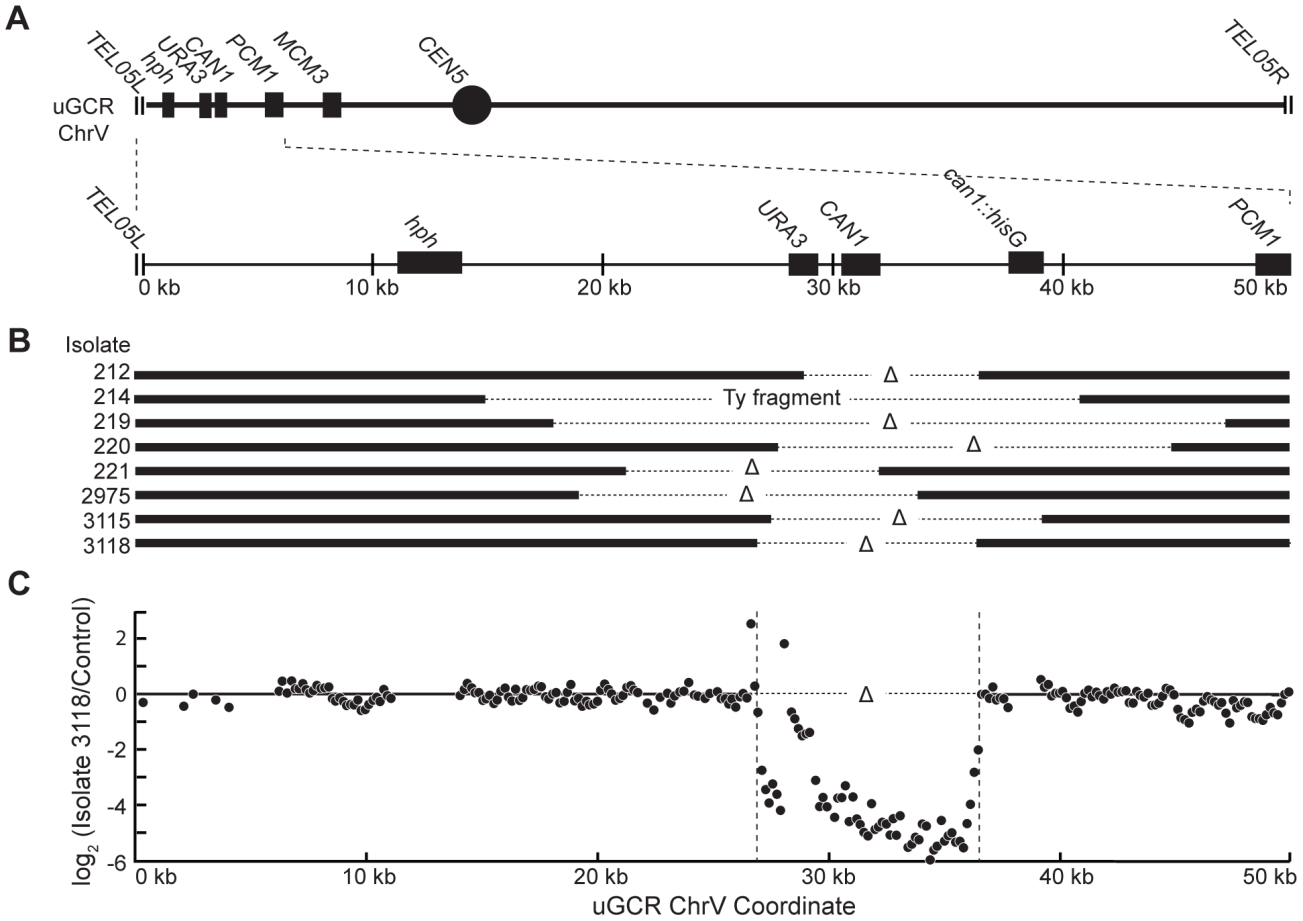
*hph*+ GCRs associated with wild-type-sized chrV are interstitial deletions. (**A**) Diagram of the uGCR chrV and the features on the first 50 kb containing *hph*, the *CAN1/URA3* cassette and the GCR breakpoint region. (**B**) Map of the retained (solid bar) and deleted (dotted line) regions for the 8 *hph*+ GCR isolates with wild-type-sized chrV. Interstitial deletions on chrV entirely (isolates 214, 219, 220, 2975, 3115, and 3118) or partially (isolates 212 and 221) spanned the *CAN1/URA3* cassette. All of the isolates are simple deletions, indicated by a Δ symbol, other than 214, which is fused to a fragment of a Ty element. (**C**) The log base 2 ratio of the aCGH hybridization intensity for a portion of chrV L from isolate 3118 illustrating the agreement between aCGH and sequenced junctions. The coordinates are mapped to the “uGCR Chromosome V” of RDKY6677, which differs somewhat from the database S288c sequence due to modifications introduced onto chrV during strain construction. No data are present for the *hph* and *can1::hisG* insertions because these regions were not probed by the aCGH array.

Analysis of the 8 *hph*+ GCRs with a wild-type-sized chrV revealed that they all contained interstitial deletions. We used PCR to map and amplify the rearrangement breakpoints. Sanger sequencing of the PCR products revealed the presence of interstitial deletions that spanned the *CAN1/URA3* cassette (Figure 3A and B) and had short sequence identities at the breakpoint junctions (0–5 basepairs in length; Figure S2), consistent with previous observations [18]. In addition, isolate 214 contained an insertion of a ∼4 kb fragment of a Ty retrotransposon at the breakpoint. Lack of copy number changes other than the interstitial deletion was verified by array comparative genomic hybridization (aCGH) of isolate 3118 (Figure 3C). Paired-end whole genome sequencing (WGS) of isolate 3118 (Table S1 and S2) confirmed the interstitial deletion by the identification of 572 read pairs (‘junction-defining’ read pairs) that had mapped inter-read distances of ∼5.4 kb as compared to the median mapped inter-read distance of 417 bp for all 11,333,616 uniquely mapping read pairs (Figure S3). Additionally, alignment of 114 unmapped reads, which were paired with a read that mapped adjacent to the junction-defining read pairs (‘junction-sequencing’ reads), identified the same junction sequence observed by PCR amplification and Sanger sequencing (Figure S3).

**Figure 4:**
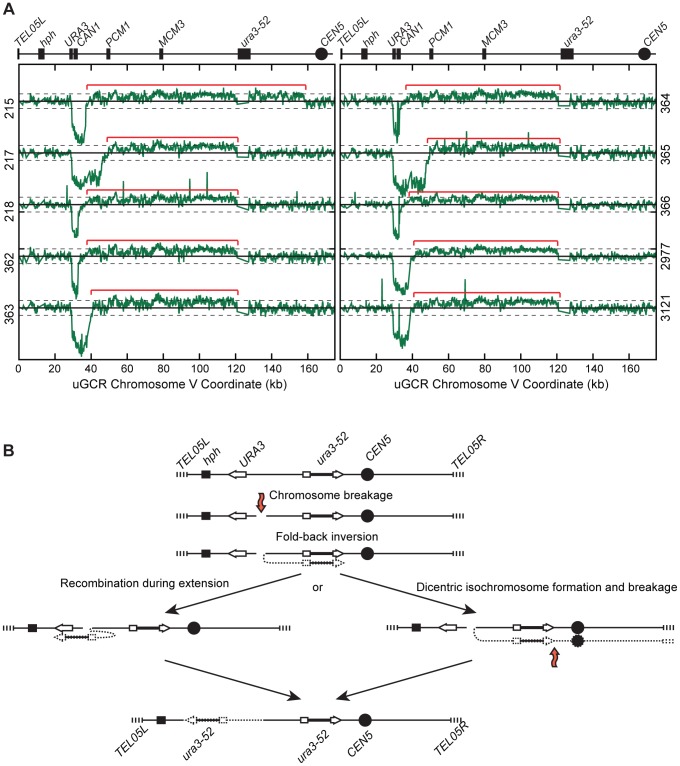
*hph*+ GCRs associated with chrV larger than wild-type contain duplicated chrV sequences. (**A**) The log base 2 ratio of the aCGH hybridization intensity for chrV L of *hph*+ isolates with larger than wild-type-sized chrV. The solid horizontal bar is at 0 and dashed lines are at −1 and 1 (2-fold decreased and increased, respectively). Probes were mapped onto the “uGCR Chromosome V” coordinate system. Chromosomal features such as *hph*, the *CAN1/URA3* cassette, the *ura3-52* mutation, and the centromere (*CEN5*) are indicated at top. Red brackets indicate duplicated chromosomal regions that span from the GCR breakpoint region (between the *CAN1/URA3* cassette and *PCM1*) to a Ty-related element, most frequently *ura3-52*. (**B**) Proposed mechanism for rearrangement formation (see Discussion). Orange arrows indicate DSBs.

The 10 *hph*+ GCRs with a large chrV were inverted duplications associated with a second homology-mediated rearrangement. aCGH analysis revealed that in the strains containing these GCRs all of the copy number changes detected were restricted to chrV: the changes associated with these GCRs included a ∼4–19 kb chrV L deletion spanning the *CAN1/URA3* cassette, and a ∼80–100 kb chrV L duplication extending from the GCR breakpoint region, which is bounded by the *CAN1/URA3* cassette and *PCM1* (Figure 1A), to a centromeric repetitive element, which was most frequently the Ty-containing *ura3-52* (Figure 4A). In each case, the aCGH data also indicated that the GCRs retained the *hph*-containing region of chrV from *TEL05L* to the telomeric half of *YEL068C*, consistent with HR-mediated fusion between *ura3-52* and *URA3* in the *CAN1/URA3* cassette. We verified the *ura3-52/URA3* fusion by PCR amplification and Sanger sequencing (Figure S4). WGS of 8 isolates (Table S1) identified and sequenced an inversion junction at the telomeric end of the chrV L duplication (Figure S5; Table S2). If these junctions were formed by folding back and priming of a single strand (Figure 4B), then the homologies for priming were 3–9 bases and the unpaired single-stranded hairpin ranged from 25 to 44 bases (Figure S5). Palindromes are typically difficult to amplify, which may explain the reduced number of junction-defining read pairs recovered for the inversion junctions relative to other rearrangement junctions introduced during strain construction (Table S2). The analyses of these inverted duplication GCRs were consistent with the changes observed by PFGE (Figure 2B and C; Figure S1), because the duplicated regions lacked *Asc*I sites, and the rearranged chromosomes were capped by the *Asc*I-containing left telomeric fragment.

### Some *hph*− rearrangements isolated using the *tel1Δ* uGCR assay are inverted duplications

**Figure 5:**
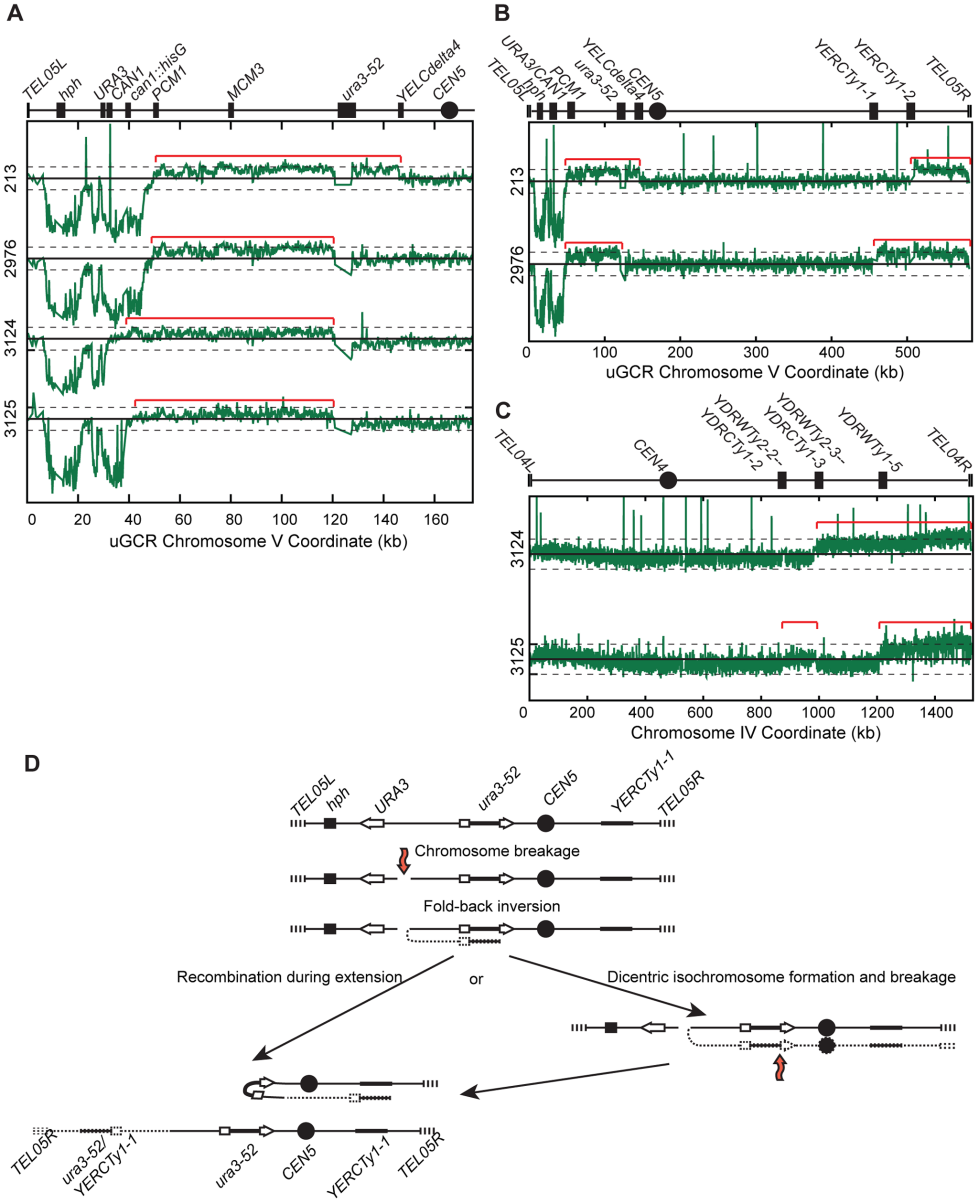
*hph*− GCRs associated with chrV larger than wild-type contain duplicated chrV sequences. (**A**) The log base 2 ratio of the aCGH hybridization intensity for chrV L for *hph*− isolates with chrV larger than wild-type. The solid horizontal bar is at 0 and dashed lines are at −1 and 1 (2-fold decreased and increased, respectively). Probes were mapped onto the “uGCR Chromosome V” coordinate system. Chromosomal features such as *hph*, the *CAN1/URA3* cassette, the *ura3-52* mutation, and the centromere (*CEN5*) are indicated at top. Red brackets indicate duplicated chromosomal regions that span from the GCR breakpoint region (between the *CAN1/URA3* cassette and *PCM1*) to a Ty-related element, most frequently *ura3-52*. (**B**) The log base 2 ratio of aCGH hybridization intensity for all of chrV for isolates 213 and 2976. Red brackets indicate duplicated chromosomal regions. (**C**) The log base 2 ratio of aCGH hybridization intensity for all of chrIV for isolates 3124 and 3125. Red brackets indicate duplicated chromosomal regions. (**D**) Proposed mechanism for rearrangement formation (see Discussion). Orange arrows indicate DSBs.

PFGE analysis of the 13 *hph*− GCR-containing isolates from the *tel1Δ* uGCR assay strain revealed that 9 contained a wild-type-sized chrV and 4 contained a large chrV (Figure S6A). PCR mapping [26] revealed that the 9 isolates with wild-type-sized chrV had deletions that included the *CAN1/URA3* cassette; sequencing the breakpoints of 4 of these GCRs confirmed that one was a translocation and 3 were *de novo* telomere additions (Figure S6B). In contrast, aCGH analysis of the 4 isolates with a larger than wild-type chrV (Figure 5A–C) was consistent with a chrV inverted duplication combined with rearrangements targeting homologies unrelated to *URA3*: these GCRs contained a chrV L deletion from the telomere to the GCR breakpoint region (Figure 5A), a chrV L duplication from the GCR breakpoint region to a Ty-related repetitive element (Figure 5A), and an additional duplication of at least one other additional genomic region bounded by Ty-related elements and telomeres (Figure 5B and C). Isolate 3125 had two duplicated regions (between the inverted Ty pairs *YDRWTy2-2/YDRCTy1-2* and *YDRWTy2-3/YDRCTy1-3* and between *YDRWTy1-5* and *TEL04R*), which was consistent with a mechanism involving more than one round of HR-mediated rearrangements similar to GCRs obtained using other GCR assays [15], [20]. The inversion junctions were identified and sequenced by analysis of WGS data from isolates 3124 and 3125 (Figure S5; Table S1 and S2). Thus, the *hph*− inverted duplications differed from the *hph*+ inverted duplications only with regard to the homologies involved in the resolution of the initial inversion chromosome (Figure 5D).

### Detection of chrV L duplications by a multiplex ligation-mediated probe amplification (MLPA) assay

Because inverted duplications could form with or without *hph* retention, we developed an MLPA probe set [27], [28] to identify chrV L duplications. MLPA results were validated by comparison with aCGH data for isolates 213, 217, 362, and 3178 (Figure S7). Using MLPA we verified that the 9 *hph*− GCRs with a wild-type sized chrV from the *tel1Δ* uGCR assay strain lacked a chrV L duplication. The aggregate data indicated that 14 of 31 GCRs isolated in the *tel1Δ* uGCR assay strain contained chrV L duplications consistent with inverted duplications (Table 1), whereas the remaining 17 GCRs lacked chrV L duplications and were consistent with interstitial deletions, *de novo* telomere additions, or translocations.

**Table 1:** Count of GCR events with and without duplication of the left arm of chromosome V from the uGCR assay.

*Genotype*	*Isolates analyzed* *	*With ChrV L duplication*			*Without ChrV L duplication*		
		*total*	*hph+*	*hph−*	*total*	*hph+*	*hph−*
Wild-type	27	1	1	0	26	1	25
*tel1*	31	14	10	4	17	8	9
*tel1 lig4*	58	27	17	10	31	1	30
*tel1 rad52*	34	0	0	0	34	5	29
*sae2*	28	21	14	7	7	0	7
*tel1 sae2*	22	11	5	6	11	1	10
*exo1 sae2*	20	5	3	2	15	1	14
*mre11*	21	1	1	0	20	1	19
*mre11-3* **	6	0	0	0	6	3	3
*mre11-H125N* **	7	0	0	0	7	3	4

* The presence of a duplication of the left arm of chromosome V was determined by WGS, aCGH, and/or MLPA.

** The *mre11-3* and *mre11-H125N* alleles were present on plasmids in a strain with a chromosomal deletion of *MRE11*.

### NHEJ is required for efficient formation of *hph*+ interstitial deletions

To investigate the mechanisms of GCR formation, we first tested the effect of a *lig4*Δ mutation, which causes an NHEJ defect [29]. In the dGCR assay, *lig4*Δ caused a modest increase in GCR rate (Table 2), and the *tel1Δ lig4Δ* double mutation caused a higher rate in the dGCR assay relative to each single mutation (p = 0.0003 and p = 0.0016, respectively, Mann-Whitney test). The *lig4*Δ mutation did not affect the GCR rate or *hph*+ retention in the uGCR assay, but the *tel1Δ lig4Δ* double mutation modestly decreased the GCR rate and increased the frequency of *hph* retention relative to the wild-type strain (p = 2×10^−8^, G-test). The most striking change in the uGCR product spectrum of the *tel1Δ lig4Δ* strain relative to the *tel1Δ* strain was the lack of GCRs that did not contain a chrV L duplication that retained *hph* (Table 1). Eighteen of 19 *hph*+ GCRs from the *tel1Δ lig4Δ* uGCR assay strain belonged to the inverted duplication class of GCRs: these GCRs had a chrV that was larger than wild-type, fusion of *ura3-52* with *URA3* (data not shown), and a chrV L duplication (Table 1). Thus, the major mechanism forming the interstitial deletion class of *hph*+ GCRs is NHEJ, which is consistent with the short homologies found at the interstitial deletion breakpoints (Figure S2).

**Table 2:** GCR rates and percent *hph* retention in *tel1*, *sae2*, and related mutants.

*Genotype*	*uGCR assay*			*dGCR assay*		
	*RDKY*	*Can^R^ 5FOA^R^ Rate* †	*hph retention*	*RDKY*	*Can^R^ 5FOA^R^ Rate* †	*hph retention*
Wild-type**	6677	2.27×10^−9^ (1)	7% (2 of 27)	6678	1.97×10^−8^ (8.7)	0% (0 of 62)
*tel1* *	6761	4.99×10^−9^ (2.2)	58% (18 of 31)§	6770	2.87×10^−8^ (13)	0% (0 of 14)
*lig4*	8012	1.53×10^−9^ (0.7)	9% (4 of 45)	8013	7.57×10^−8^ (33)	0% (0 of 28)
*lig4 tel1*	8014	<6.21×10^−10^ (0.3)	32% (19 of 59)§	8015	2.10×10^−7^ (93)	0% (0 of 14)
*rad52* *	6691	1.67×10^−8^ (7.3)	7% (2 of 27)	6708	1.09×10^−8^ (4.8)	0% (0 of 55)
*rad52 tel1*	8016	6.41×10^−8^ (28)	13% (13 of 99)	8017	1.06×10^−7^ (47)	0% (0 of 14)
*sae2* *	6737	4.23×10^−8^ (19)	50% (14 of 28)§	6754	1.65×10^−7^ (72)	0% (0 of 14)
*sae2 rad52*	8022	1.96×10^−8^ (8.6)	0% (0 of 14)	8023	2.59×10^−8^ (11)	0% (0 of 14)
*sae2 tel1*	8018	7.14×10^−8^ (31)	26% (33 of 128)§	8019	6.63×10^−8^ (29)	0% (0 of 14)
*exo1* *	6729	2.00×10^−9^ (0.9)	0% (0 of 38)	6746	8.44×10^−8^ (37)	0% (0 of 14)
*sae2 exo1*	8020	8.13×10^−9^ (3.6)	30% (16 of 54)§	8021	9.21×10^−8^ (41)	0% (0 of 14)
*sgs1* **	6687	1.69×10^−8^ (7.5)	27% (7 of 26)§	6690	1.93×10^−6^ (850)	0% (0 of 54)
*sgs1 exo1*	8032	5.70×10^−6^ (2511)	0% (0 of 164)§	8033	5.70×10^−6^ (2511)	0% (0 of 28)
*mre11* *	6686	5.75×10^−7^ (253)	9% (6 of 64)	6689	1.52×10^−6^ (670)	0% (0 of 57)
*mre11 tel1*	8154	1.50×10^−6^ (659)	7% (2 of 28)	8155	7.33×10^−6^ (3228)	0% (0 of 14)
*xrs2-11*	8156	2.25×10^−9^ (1.0)	30% (6 of 20)§	8157	1.08×10^−7^ (47)	11% (3 of 28)

* Rate data from [14].

** Rate and *hph* retention data from [14].

† Rate of accumulating Can^r^ 5FOA^r^ progeny. The number in parenthesis is the fold increase relative to the wild-type uGCR assay.

§ Retention of *hph* in GCR-containing isolates is statistically significantly different than wild type (G-test).

### HR is required for efficient formation of *hph*+ inverted duplications

Because the inverted duplication class of GCRs involved homology-mediated rearrangements, we tested the effect of a *rad52Δ* mutation that eliminates HR. In the dGCR assay, a *rad52Δ* mutation suppressed the GCR rates [14], and the *tel1Δ rad52Δ* double mutant had modestly increased GCR rates relative to both single mutants (Table 2). The *rad52Δ* and the *tel1Δ rad52Δ* mutants had higher GCR rates in the uGCR assay, but had no significant increase in the frequency of *hph* retention relative to the wild-type strain (p = 1.0 and 0.5, respectively, G-test; Table 1). Analyses of the 13 *hph*+ GCRs from the *rad52Δ tel1*Δ uGCR assay strain was consistent with these GCRs belonging to the interstitial deletion class of GCRs: these GCRs had wild-type-sized chrV, no *ura3-52/URA3* fusions (data not shown), and none of the 5 *hph*+ isolates tested by MLPA had a chrV L duplication (Table 1). These data suggest that HR mediates a key step in the formation of the inverted duplication class of GCRs, likely by the formation of stable monocentric chromosomes (Figure 4B and 5D).

### Interstitial deletions and inverted duplications are formed in the wild-type uGCR assay strain

Two of 27 GCRs formed in the wild-type uGCR assay strain retained *hph+* (isolates 3178 and 3255). The GCR in isolate 3255 was an interstitial deletion: chrV was of wild-type size with no chrV L duplication, and WGS identified an interstitial deletion (Table 1; Figure S8A, B, and D; Table S1 and S2). The GCR in isolate 3178 was an inverted duplication: chrV was larger than wild-type due to a change in the size of the central *Asc*I fragment; it contained a chrV L duplication extending from the GCR breakpoint region to *ura3-52*; a *ura3-52/URA3* breakpoint junction was present that could be amplified by PCR; and an inversion junction was present that was identified by WGS (Table 1, S1, and S2; Figure S4, S5G, and S8A–C). The remaining 25 *hph*− GCRs formed in the wild-type uGCR assay strain were *hph*− GCRs that lacked chrV L duplications (Table 1). Thus, both the interstitial deletion and inverted duplication classes of *hph*+ GCRs were observed with the wild-type uGCR assay strain, suggesting that deletion of *TEL1* changes the efficiency rather than the pathways by which these GCRs are formed.

### DNA hairpins are likely intermediates in the formation of inverted duplications

The structures of the inverted duplication GCRs were consistent with the formation of single-stranded hairpins (Figure S5), but do not rule out interchromosomal Break-Induced Replication (BIR) events occurring after DNA replication [30], [31]. Because hairpin-capped duplexes are substrates for Sae2-promoted cleavage [32]–[34], we determined the effect of deleting *SAE2* on *hph* retention in the uGCR assay. Half of the GCRs from the *sae2Δ* uGCR assay strain were *hph*+ (14 of 28 isolates; p = 2×10^−9^, G-test). All 14 *hph*+ isolates and 7 of 14 *hph*− isolates contained GCRs that were consistent with the inverted duplication class of GCRs: all had a chrV that was larger than wild-type and had chrV L duplications as measured by MLPA (Table 1). Additionally, 13 of the 14 *hph+* isolates had a *URA3/ura3-52* fusion (data not shown). These results support the hypothesis that the formation of the inverted duplication class of GCRs involves a DNA hairpin intermediate. Deletion of *RAD52* eliminated the *sae2Δ*-mediated increase in the frequency of *hph*+ GCRs in the uGCR assay (Table 2), which is consistent with the importance of *RAD52* for the HR-dependent event that occurs after inversion formation and stabilizes the inverted duplication GCRs formed in the *tel1Δ* uGCR assay strain.

### *TEL1* promotes the formation of the *hph+* inverted duplication class of GCRs in *sae2Δ* mutants

Mec1 and Tel1 promote Sae2 activity by phosphorylation [35]–[37]. To test if *TEL1* and *SAE2* function in the same pathway in the formation of *hph*+ GCRs, we generated *tel1Δ sae2Δ* double mutant strains. In the dGCR assay, the double mutant had a 2.5-fold lower GCR rate relative to the *sae2Δ* single mutant (p = 0.0001, Mann-Whitney test) and a 2.3-fold higher GCR rate relative to the *tel1Δ* single mutant (p = 0.006; Table 2). In the uGCR assay, the double mutant had a 1.7-fold higher GCR rate relative to the *sae2Δ* single mutant, and the frequency of *hph* retention was lower than seen in both the *tel1Δ* and *sae2Δ* single mutant strains (p = 1×10^−13^ and p = 2×10^−8^, respectively, G-test), but still was higher than wild-type (p = 2×10^−10^; Table 2). MLPA analysis of *hph*+ and *hph*− GCRs from the *tel1Δ sae2Δ* uGCR assay strain revealed that 50% (11 of 22) contained chrV L duplications, and the frequency of *hph*+ GCRs without chrV L duplications (probable interstitial deletions), like the case of the *sae2Δ* strain, was much lower than seen with the *tel1Δ* strain (Table 1). These data suggest that *TEL1* is not required for the formation of chrV L inverted duplications in the *sae2Δ* uGCR assay strain, but does promote the formation of chrV L inverted duplications associated with *hph* retention.

### Mutations affecting Sae2 phosphorylation sites do not cause increased *hph* retention

We then tested the ability of different plasmid-borne phosphorylation-defective alleles of *SAE2* to complement the *sae2Δ* mutation (Table 3). In the dGCR assay, the *sae2^1–9^* and *sae2^2,4,5,8,9^* alleles, which eliminated multiple Mec1 and Tel1 phosphorylation sites [35], either did not or partially suppressed the increased GCR rate caused by deleting *SAE2*. In the uGCR assay, these *sae2* alleles partially complemented the increased GCR rate and decreased the *hph* retention observed in the *sae2Δ* single mutant. Sae2 is also phosphorylated by the Cdc28 cyclin-dependent kinase at Ser267, and the *sae2-S267A* mutation is phenotypically similar to a *sae2Δ* single mutant [38]. The *sae2-S267A* allele did not suppress the higher GCR rate of the *sae2Δ* mutation in either GCR assay; however, the *sae2-S267A* allele did not cause increased *hph* retention in the uGCR assay. The lack of *hph* retention in strains containing these *sae2* phosphorylation-defective alleles suggests that these alleles are not simply null mutations but additionally disrupt *hph* retention potentially by affecting Tel1 signaling or by affecting the capture of the acentric *hph*-containing fragment.

**Table 3:** GCR formation in plasmid-complemented strains.

*Genotype*		*uGCR Assay*		*dGCR Assay*	
*Strain*	*Plasmid*	*Can^R^ 5FOA^R^ rate* †	*hph retention*	*Can^R^ 5FOA^R^ rate* †	*hph retention*
*sae2*	*SAE2*	1.10×10^−9^ (0.5)	20% (3 of 15)	8.71×10^−8^ (38)	0% (0 of 13)
*sae2*	empty	8.76×10^−8^ (39)	45% (9 of 20)§	1.99×10^−7^ (88)	14% (4 of 24)
*sae2*	*sae2^1–9^*	1.87×10^−8^ (8.3)	24% (5 of 21)	2.02×10^−7^ (89)	0% (0 of 33)
*sae2*	*sae2^2,4,5,8,9^*	1.91×10^−8^ (8.4)	14% (3 of 21)	1.06×10^−7^ (47)	0% (0 of 31)
*sae2*	*sae2-S267A*	6.79×10^−8^ (30)	15% (3 of 20)	1.79×10^−7^ (79)	0% (0 of 13)
*mre11*	*MRE11*	8.14×10^−10^ (0.4)	15% (3 of 20)	1.34×10^−7^ (59)	0% (0 of 14)
*mre11*	empty	1.33×10^−6^ (586)	3% (2 of 58)	4.85×10^−6^ (2134)	0% (0 of 14)
*mre11*	*mre11-2*	1.17×10^−6^ (516)	4% (2 of 45)	3.43×10^−6^ (1512)	0% (0 of 14)
*mre11*	*mre11-3*	5.03×10^−7^ (221)	8% (3 of 40)	1.36×10^−6^ (598)	0% (0 of 14)
*mre11*	*mre11-H125N*	2.36×10^−7^ (104)	10% (6 of 60)	4.88×10^−7^ (215)	0% (0 of 14)
*mre11*	*mre11S*	1.78×10^−8^ (7.7)	8% (4 of 49)	1.15×10^−7^ (64)	0% (0 of 10)

† Rate of accumulating Can^r^ 5FOA^r^ progeny. The number in parenthesis is the fold increase relative to the wild-type uGCR assay.

§ Retention of *hph* in GCR-containing isolates is statistically significantly different than wild type (G-test).

### Disruption of the Tel1 interaction with Mre11-Xrs2-Rad50, but not other Mre11 defects, causes increased *hph* retention

Tel1 and Sae2 interact functionally with the Mre11-Rad50-Xrs2 (MRX) complex [39], [40]. However, the *mre11Δ* single mutation caused increased GCR rates in both GCR assays without affecting the frequency of *hph*-retention in the uGCR assay (Table 2). In addition, the *mre11Δ* uGCR strain did not have an increased frequency of *hph*− GCRs associated with chrV L duplications (Table 1). Consistent with the differences in rate and types of GCRs formed, the *tel1Δ* and *mre11Δ* mutations were not epistatic; the *tel1Δ mre11Δ* double mutant had higher GCR rates relative to both single mutants in both GCR assays and did not have increased *hph* retention in the uGCR assay, similar to the *mre11Δ* single mutant (Table 2). These results suggest that Mre11 plays roles in maintaining genome stability that are independent of Tel1 and Sae2.

The stability of the MRX complex is more important than the Mre11 nuclease function in maintaining genome stability [41], [42]; however, the nuclease-defective *mre11-D56N* and *mre11-H125N* alleles are similar to the *sae2Δ* mutation in causing persistent Mre11 foci and in reducing recombination at inverted repeats [33], [43]. We therefore investigated if nuclease-defective *mre11* alleles might increase *hph* retention in the uGCR assay like the *sae2Δ* mutation. Plasmid-borne wild-type *MRE11* and the meiotic-processing defective *mre11S* allele [44] complemented or largely complemented the *mre11Δ* defect, respectively, in both GCR assays (Table 3). In contrast, the *mre11-2* allele, which causes defects in MRX complex formation [45], caused defects similar to those caused by the *mre11Δ* mutation, and the nuclease-defective *mre11-3* and *mre11-H125N* alleles [45], [46] caused partial defects. None of the tested *mre11* alleles tested significantly changed the frequency of *hph*-retaining GCRs in the uGCR assay relative to the wild-type or *mre11Δ* mutant strains (Table 3), and all of the GCRs analyzed from the nuclease-defective *mre11* alleles lacked inverted duplications (Table 1). Thus, the GCRs accumulating in strains with a *sae2Δ* mutation differ from GCRs in the *mre11Δ* and nuclease-defective *mre11* mutations, despite the similarity of these mutations in assays for inverted repeat-mediated recombination likely caused by defects in hairpin cleavage [33]. The differences in types and rates of GCRs formed in strains with *mre11* mutations indicate that defects in *MRE11* cause additional defects relative to defects in *SAE2* and that the GCRs that are initially formed in these *mre11* strains are not mediated by hairpin-mediated formation of inverted duplications.

Tel1 is recruited to DSBs through interaction with the C-terminus of Xrs2, and consequently the *xrs2-11* allele, which encodes a truncated Xrs2 protein lacking the C-terminal 162 residues that does not interact with Tel1, is similar to a *tel1Δ* mutation in some assays [39]. The *xrs2-11* mutant had an increased GCR rate in the dGCR assay that was 4- to 5-fold higher than that of the wild-type and *tel1Δ* strains (Table 2). In contrast, the GCR rate in the uGCR assay in the *xrs2-11* mutant was not distinguishable from that of the wild type or *tel1Δ* strains, whereas the frequency of *hph* retention in the *xrs2-11* uGCR assay strain was increased relative to wild-type (p = 0.003; G-test) but not significantly different from that caused by the *tel1Δ* mutation. These data suggest that Tel1 recruitment to the MRX complex is required to suppress the formation of *hph*-retaining GCRs, despite the fact that other MRX defects cause higher GCR rates without increasing *hph* retention.

### End resection promotes GCRs associated with chrV L duplications and *hph* retention

End resection during double strand break repair (DSB) repair in *S. cerevisiae* is proposed to involve two steps [47]–[49]: the initial removal of a short oligonucleotide by the MRX complex in conjunction with Sae2 followed by extensive resection by either Exo1 alone or by Sgs1 in combination with Dna2. Deletion of both *SAE2* and *SGS1* causes synthetic lethality, which can be suppressed by deleting *YKU70* [50], but the *sae2Δ* mutation is not lethal in combination with an *exo1Δ* mutation. In the uGCR assay, the *sae2Δ exo1Δ* double mutant had a level of GCRs retaining *hph* and having a chrV L duplication that was intermediate between that of the wild-type and *sae2Δ* strains (Tables 1 and 2). Additionally, the double mutant had modestly reduced GCR rates relative to the *sae2Δ* single mutant in both assays (p = 0.002 uGCR assay, p = 0.06 dGCR assay; Mann-Whitney) (Table 2). Elimination of both resection pathways in the *sgs1Δ exo1Δ* double mutant caused a substantial increase in GCR rate relative to the single mutants in both GCR assays (Table 2). The fact that the *sgs1Δ exo1Δ* double mutant had the same GCR rate in both GCR assays is consistent with the observation that the *sgs1Δ exo1Δ* double mutant repairs DSBs primarily through the formation of *de novo* telomeres [51] and with the significantly reduced frequency of *hph* retention in the uGCR assay (Table 2; p = 5×10^−7^, G-test). Together these results suggest that at least Exo1 contributes to the formation of *hph*-retaining chrV L inverted duplications in *sae2Δ* mutants, potentially by mediating resection to initiate a DNA hairpin structure.

### Retention of *hph* in GCRs formed in checkpoint-defective mutant strains

Because *TEL1* is involved in DNA damage checkpoint signaling [52]–[54], we analyzed other mutations affecting the DNA damage and replication checkpoints (Table 4). Most of the mutations tested did not cause increased frequency of *hph* retention in the uGCR assay, except for *rad53Δ sml1Δ*, *tof1Δ*, and *mrc1Δ tof1Δ*. The *hph+* GCRs obtained from the *rad53Δ sml1Δ* strain were primarily inverted duplications (11 of 13) and the *hph*+ GCRs from the *mrc1Δ tof1Δ* double mutant were primarily interstitial deletions (8 of 10) on the basis of the size of the rearranged chrV and the presence of a *URA3/ura3-52* fusion junction detected by PCR (data not shown). Thus, the defects in *tel1Δ* mutants appear to be distinct from defects causing increased *hph* retention in the *mrc1Δ tof1Δ* or *rad53Δ sml1Δ* mutants.

**Table 4:** GCR rates and percent *hph* retention in checkpoint defective mutants.

Genotype	*uGCR assay*			*dGCR assay*		
	*RDKY*	*Can^R^ 5FOA^R^ Rate* †	*hph retention*	*RDKY*	*Can^R^ 5FOA^R^ Rate* †	*hph retention*
Wild-type**	6677	2.27×10^−9^ (1)	7% (2 of 27)	6678	1.97×10^−8^ (8.7)	0% (0 of 62)
*tel1* *	6761	4.99×10^−9^ (2.2)	58% (18 of 31)§	6770	2.87×10^−8^ (13)	0% (0 of 14)
*mec1 sml1* *	6760	2.34×10^−8^ (10)	2% (2 of 101)§	6769	1.50×10^−7^ (66)	0% (0 of 13)
*rad53 sml1* *	6762	5.60×10^−8^ (25)	31% (13 of 42)§	6771	3.05×10^−7^ (134)	0% (0 of 14)
*dun1* **	6763	1.63×10^−8^ (7.2)	0% (0 of 13)	6772	1.61×10^−7^ (71)	0% (0 of 14)
*chk1* **	6764	1.76×10^−8^ (7.8)	0% (0 of 22)	6773	1.96×10^−7^ (86)	0% (0 of 61)
*rad24* **	6759	2.00×10^−8^ (8.8)	0% (0 of 13)	6768	1.97×10^−7^ (87)	0% (0 of 13)
*mrc1* *	6730	3.35×10^−9^ (1.5)	19% (5 of 26)	6747	3.75×10^−7^ (165)	0% (0 of 14)
*mrc1-aq* **	6766	1.51×10^−9^ (0.7)	0% (0 of 7)	6775	1.23×10^−7^ (54)	0% (0 of 7)
*tof1* *	6767	5.71×10^−9^ (2.5)	33% (7 of 21)§	6776	4.25×10^−7^ (187)	0% (0 of 14)
*mrc1 tof1* **	6779	6.41×10^−8^ (28)	64% (9 of 14)§	6780	1.26×10^−6^ (555)	0% (0 of 14)
*mrc1-aq tof1* **	6848	3.69×10^−9^ (1.6)	0% (0 of 11)	6849	2.06×10^−7^ (91)	0% (0 of 14)
*hta-S129X* ‡	8010	4.61×10^−10^ (0.2)	12% (1 of 8)	8011	6.63×10^−8^ (29)	0% (0 of 7)

* Rate data from [14].

** Rate and *hph* retention data from [14].

† Rate of accumulating Can^r^ 5-FOA^r^ progeny. The number in parenthesis is the fold increase relative to the wild-type *yel068c::CAN1/URA3* assay.

‡ *hta-S129X* is the genotype *hta1-S129X hta2-S129X*.

§ Retention of *hph* in GCR-containing isolates is statistically significantly different than wild type (G-test).

### Retention of *hph* in GCRs formed in strains containing mutations affecting *de novo* telomere addition

Strains with *tel1* mutations have short telomeres and can form *de novo* telomere additions, even if the efficiency appears to be decreased in some cases [16], [17], [22], [55]. Because efficient *de novo* telomere addition might be predicted to prevent the formation of both interstitial deletion and inverted duplication GCRs, we investigated strains with mutations affecting *de novo* telomere addition (Table 5). To test mutations affecting telomerase, we generated post-senescent type II survivor *est1Δ* and *est3Δ* strains after sporulating heterozygous *est1Δ/EST1* or *est3Δ/EST3* diploids. These strains had an increased frequency of *hph* retention in the uGCR assay (p = 0.0006 and p = 2×10^−9^, respectively, G-test; Table 5). Similarly, deletion of *YKU70* and *YKU80*, which are required for *de novo* telomere addition and NHEJ but not telomere maintenance [29], [56], increased the frequency of *hph* retention to 44% and 40%, respectively (p = 7×10^−5^ and p = 5×10^−5^, respectively, G-test; Table 5). In contrast, deletion of *LIG4*, which is required for NHEJ but not *de novo* telomere addition [29], [56], did not increase the frequency of *hph* retention (Table 2).

**Table 5:** GCR rates and percent *hph* retention in mutants affecting *de novo* telomere addition.

Genotype	*uGCR*			*dGCR*		
	*RDKY*	*Can^R^ 5FOA^R^ rate* †	*hph retention*	*RDKY*	*Can^R^ 5FOA^R^ rate* †	*hph retention*
Wild-type**	6677	2.27×10^−9^ (1)	7% (2 of 27)	6678	1.97×10^−8^ (8.7)	0% (0 of 62)
*tel1* *	6761	4.99×10^−9^ (2.2)	58% (18 of 31)§	6770	2.87×10^−8^ (13)	0% (0 of 14)
*est1*	8000	<1.01×10^−9^ (<0.5)	33% (7 of 21)§	8001	1.96×10^−8^ (8.7)	3% (1 of 37)
*est3*	8002	<9.50×10^−10^ (<0.4)	58% (11 of 19)§	8003	1.85×10^−8^ (8.1)	0% (0 of 14)
*yku70*	8004	<5.32×10^−10^ (<0.2)	44% (7 of 16)§	8005	5.33×10^−8^ (23)	0% (0 of 14)
*yku80*	8006	<6.88×10^−10^ (<0.3)	40% (8 of 20)§	8007	2.73×10^−8^ (12)	0% (0 of 14)
*mec1 sml1* *	6760	2.34×10^−8^ (10)	2% (3 of 133)§	6769	1.50×10^−7^ (66)	0% (0 of 13)
*pif1* ***	6894	3.73×10^−7^ (164)	0% (0 of 86)§	6936	3.61×10^−7^ (159)	0% (0 of 14)
*pif1 tel1*	8008	1.52×10^−6^ (669)	0% (0 of 14)	8009	1.43×10^−6^ (629)	0% (0 of 14)

* Rate data from [14].

** Rate and *hph* retention data from [14].

*** Rate data from [21].

† Rate of accumulating Can^r^ 5-FOA^r^ progeny. The number in parenthesis is the fold increase relative to the wild-type *yel068c::CAN1/URA3* assay.

§ Retention of *hph* in GCR-containing isolates is statistically significantly different than wild type (G-test).

Consistent with the effects of mutations eliminating *de novo* telomere addition, mutations that increase the rate of *de novo* telomere addition caused a reduced frequency of *hph* retention. Deletion of *MEC1*, which causes increased rates of GCRs that are mediated primarily by *de novo* telomere addition due to loss of inhibition of *CDC13* [22], [57], simultaneous deletion of *SGS1* and *EXO1*, which results in high rates of healing of DSBs by *de novo* telomere addition [51], and deletion of *PIF1*, which causes increased rates of GCRs mediated primarily by *de novo* telomere addition due to loss of inhibition of telomerase at sites of *de novo* telomere addition [16], [58]–[60], caused a significantly reduced frequencies of retention of *hph* relative to that of the wild-type strain in the uGCR assay (*mec1Δ sml1Δ* p = 0.008; *sgs1Δ exo1Δ* p = 5×10^−7^; *pif1Δ* p = 0.0002; Table 2 and 5). In addition, *hph*+ GCRs were not observed in the *pif1Δ tel1Δ* double mutant, reminiscent of the inability of *tel1Δ* to suppress the increased GCR rate caused by *de novo* telomere addition in the *pif1-m2* mutant [16]. Consistent with the hypothesis that *de novo* telomere additions were the primary type of GCR formed in the *pif1Δ tel1Δ* double mutant strains, the rates in the uGCR and dGCR assays were essentially the same, like that seen in the *pif1Δ* single mutant and the *sgs1Δ exo1Δ* double mutant (Table 2 and 5). Together, these results suggest that *de novo* telomere addition suppresses *hph* retention, potentially by competing for broken ends that could otherwise undergo either NHEJ or resection leading to interstitial deletions or inverted duplications.

## Discussion

The observed genotype-specific increase in retention of the telomeric *hph* marker in the uGCR assay was due to the formation of NHEJ-dependent interstitial deletions spanning the *CAN1/URA3* cassette or inverted duplications that recaptured the telomeric end of chrV using the homology between *URA3* of the *CAN1/URA3* cassette and *ura3-52* located on chrV L. Previous studies identified inverted duplication GCRs involving dicentric isoduplication intermediates; however, these occurred at low rates [18] and were difficult to identify, because their identification required sequencing of their rearrangement breakpoints. The ability of the uGCR assay to capture these events combined with more facile product analysis provided a convenient genetic assay for use in studying the structural features and genetic requirements underlying these types of GCRs. The *tel1Δ* uGCR assay strain had increased frequencies of forming both types of *hph*+ GCRs, whereas other mutant backgrounds with increased *hph* retention yielded primarily interstitial deletions (*mrc1Δ tof1Δ*) or inverted duplications (*sae2Δ* and *rad53Δ sml1Δ*). Remarkably, both types of rearrangements were observed in GCRs formed in the wild-type uGCR assay strain. These *hph*-retaining GCRs were suppressed by *de novo* telomere addition; mutations promoting *de novo* telomere addition (*mec1Δ*, *pif1Δ*, and *sgs1Δ exo1Δ*) suppressed *hph* retention, whereas mutations inhibiting *de novo* telomere addition (*est1Δ*, *est3Δ*, *yku70Δ*, and *yku80Δ*) enhanced *hph* retention. In contrast, extensive competition between other implicated pathways likely precludes simple extrapolation of the conclusions based on the phenotypes caused by individual mutations that result in increased frequencies of *hph*-retaining GCRs to the predicted effects of other mutations affecting the same or related pathways. Examples include the differences between *tel1Δ* and *mec1Δ*, between *tel1Δ* and *rad53Δ*, between *tel1Δ* and *mre11Δ*, between *sae2Δ* and *mre11Δ*, as well as the differences between mutations affecting different features of Mre11-Rad50-Xrs2. Despite this, our analysis allowed us to link an unusual signature of GCRs to specific genetic defects.

Current and previous results suggest that several mechanisms contribute to the *hph*+ GCRs observed. The GCRs could be initiated by one or more DSBs between *hph* and *PCM1*, the most telomeric essential gene, although a DSB-independent mechanism for generating similar products has been proposed for forks stalling in the context of large inverted repeats [61], [62]. Interstitial deletions then appear to be formed by NHEJ-mediated rejoining of the two ends associated with potential processing of the ends at the DSB in some cases. Inverted duplications appear to be initiated by 5′ resection of a DSB followed by fold-back invasion of the 3′ single stranded end (Figure S5 and S9A). Subsequently, one of three mechanisms operate (Figure S9B): 1) intramolecular BIR occurs up to the position of *ura3-52* followed by HR-mediated template switching to the telomeric *URA3* and continuation of BIR to the end of chrV; 2) intermolecular BIR extends the entire length of chrV yielding an isoduplication chromosome that then breaks during cell division and is resolved by secondary rearrangements to yield a stable monocentric chromosome [63]; and 3) the fold-back hairpin is covalently closed followed by replication to yield an isoduplication chromosome, which is further processed as described above in mechanism 2. Notably, *hph+* inverted duplications were much more prevalent than *hph*− inverted duplications resolved by HR between a chrV Ty element and any of the other 254 Ty related elements in the genome. Strand switching during BIR (mechanism 1; [64], [65]) combined with the possibility that the telomeric *hph+* fragment is recombinogenic because it contains a DSB could explain this bias. However, if an isoduplication chromosome is formed first (mechanisms 2 and 3), it must subsequently break during cell division before undergoing a secondary rearrangement(s) to capture a new telomere. Consequently, the telomeric *hph*-containing fragment might be diluted out by loss or segregation into the wrong progeny during cell division, thereby reducing the formation of *hph+* recombinants; this would allowing other Ty-related sequences to serve as substrates for HR with the broken isoduplication chromosome at higher relative efficiencies relative to the *hph*-containing fragment. Together, these models predict that genetic alterations that either directly or indirectly facilitate hairpin formation, protect hairpins that have formed, promote the use of the *hph*-containing fragment as a template, facilitate NHEJ, or suppress pathways that compete with these events will increase the formation of the types of *hph*+ GCRs seen in the present study.

The *sae2Δ* and *tel1Δ* uGCR assay strains accumulated high frequencies of *hph*+ inverted duplications that required HR for their formation. However, these results are not consistent with a simple model in which *hph+* inverted duplications are suppressed primarily by Tel1-activated Sae2, which is phosphorylated by Tel1 and Mec1 [35], because *sae2* phosphosite mutations did not caused increased levels of *hph*+ GCRs in the uGCR assay, the *tel1Δ* mutant uGCR assay strain differed from the *sae2Δ* strain by accumulating interstitial deletion GCRs in addition to inverted duplication GCRs, and the *tel1Δ sae2Δ* double mutant uGCR assay strain had suppressed levels of *hph+* GCRs relative to both single mutants. The lack of interstitial deletion GCRs in the *sae2Δ* uGCR assay strain would be consistent with Sae2 primarily promoting cleavage of DNA hairpins [33], [34], [66] and with Tel1 affecting multiple pathways, potentially including promotion of *de novo* telomere additions, suppression of NHEJ, and/or suppression of hairpin cleavage by Sae2-MRX. In addition, GCRs formed in the *tel1Δ sae2Δ* uGCR strain do have higher levels of *hph−* GCRs, indicating that the retention or preferential use of the acentric *hph*-containing telomeric chrV fragment during BIR is dependent on Tel1 in the absence of Sae2. Consistent with this model, *TEL1* can more readily compensate for the deletion of *MEC1* in strains with *sae2Δ* mutations [54], presumably because of increased Tel1 signaling from DSBs that are not resected due to the uncleaved terminal hairpins that accumulate in *sae2Δ* mutants. Thus, our data would suggest that the *sae2* phosphosite mutations, unlike a *sae2Δ* mutation, may disrupt functions of Tel1 that promote use of the *hph*-containing telomeric chrV fragment during BIR. Our data, however, do not rule out a scenario in which moderate overexpression of the mutant *sae2* alleles from low copy number *ARS CEN* plasmids might be sufficient to overcome the effect of these phosphosite mutants. The fact that *TEL1* and *SAE2* jointly suppress inverted duplications suggests an alternative explanation for the apparent requirement of *TEL1* and *SAE2* in microhomology-mediated end joining (MMEJ; [67]): *TEL1* and *SAE2* may suppress pathways that compete with MMEJ for substrates rather than directly functioning in MMEJ.

Consistent with the differences in the effects of defects in *TEL1* and *SAE2* on the rate and types of GCRs formed, genetic analyses revealed that the effects of mutations affecting related pathways are difficult to predict. For example, the increased *hph* retention seen in the *rad53Δ sml1Δ* strain, which is primarily due to inverted duplications and not interstitial deletions, argues that Tel1 has additional repair-related functions that can suppress the formation of *hph*+ GCRs. However, extrapolation of this result to other DNA damage checkpoint defective mutations is problematic. For example, the *mec1Δ sml1Δ* double mutant uGCR assay strain had a significantly increased accumulation of *hph*− GCRs relative to the wild-type strain likely due to a failure to suppress *de novo* telomere additions [22], [57]. Similarly, both Tel1 and Sae2 function in conjunction with the MRX complex *in vivo*, and disruption of the Tel1-Xrs2 interaction caused increased formation of *hph*+ GCRs similar to that caused by the *tel1Δ* mutation. Thus, the recruitment of Tel1 to DSBs by MRX is likely required for suppressing *hph* retention. Yet, the *mre11Δ* mutation and *mre11* point mutations that disrupt complex formation and nuclease activity result in much higher GCR rates than the *tel1Δ* and *sae2Δ* single mutations but did not result in increased accumulation of *hph*+ GCRs in the uGCR assay, suggesting that these *mre11* mutants cause defects in addition to defects in hairpin cleavage. In sum, our results indicate that GCR signatures observed here often reflect the properties caused by individual genetic defects rather than inactivation of entire pathways in which genes of interest function, except in the case of mutations that directly affect *de novo* telomere addition; this likely limits our ability to predict the exact GCR signature caused by individual pathway defects.

Our results support the hypothesis that extensive competition between different DNA repair mechanisms determines the spectrum of genome rearrangements that accumulate in cells and this spectrum can be altered by subtle changes in the efficiencies of different pathways. This competition likely underlies the fact that rearrangement spectra caused by mutations in related genes tend to differ. Therefore, the spectrum of genome rearrangements that accumulate can provide insights into the underlying genetic defects in DNA repair pathways. For example, in a recent analysis of GCRs in human metastatic pancreatic cancers, 1 out of every 6 GCRs was a copy number change mediated by an inverted duplication that showed an association with hallmarks of telomere dysfunction and a dysregulated G1-to-S-phase transition in conjunction with an intact G2/M checkpoint [7]. These phenotypes are highly reminiscent of the phenotypes caused by defects in *TEL1* as described here and in previous studies [16], [22]–[25]. Together, these results are consistent with the notion that defects in signaling by the *ATM* pathway, which involves the human homolog of *TEL1*, may play important roles in the formation of the GCRs seen in a fraction of metastatic pancreatic cancer. Similarly, other defects such as defects in *RBBP8*, which encodes the human Sae2 homolog CtIP, might also play a role in the formation of the inverted duplications seen in metastatic pancreatic cancer. However, additional experimentation will be required to determine if the genetic insights into the origin of genome instability signatures in *S. cerevisiae* can be used to predict genetic changes with functional consequences in human cancer.

## Materials and Methods

### Construction and propagation of strains and plasmids

GCR assays were performed using derivatives of RDKY6677 (*yel068c::CAN1/URA3*) or RDKY6678 (*yel072w::CAN1/URA3*) that in addition have the genotype *MATa leu2Δ1 his3Δ200 trp1Δ63 lys2ΔBgl hom3-10 ade2Δ1 ade8 ura3-52 can1::hisG iYEL072::hph* as previously described [14]. Mutant derivatives of these strains (Table S3) were constructed using standard PCR-based gene disruption methods or mating to strains containing mutations as described [11]. The *xrs2-11* allele [39] was generated by integrating a *HIS3* marker at the 3′ end of *XRS2* to introduce a stop codon and delete the codons encoding residues 693–854 of Xrs2.

Post-senescent *est1Δ* and *est3Δ* survivors were generated by deleting one copy of either *EST1* or *EST3* in diploid versions of RDKY6677 and RDKY6678, sporulating the heterozygous diploids, and performing multiple sequential re-streaks of individual spore clones on YPD agar media until growth of the mutants was equivalent to a wild-type control as described [68]. Type I and type II survivors were distinguished on the basis of Southern blotting of *Xho*I digested genomic DNA as described [68] and by the fact that chromosomes of type I survivors do not properly enter PFGE gels [69].

Alleles of *sae2* were introduced into the *sae2::TRP1* deletion strains using pRS313-based *ARS CEN* plasmids containing the *HIS3* marker [70]. Integration plasmids bearing the *sae2^1–9^* and *sae2^2,4,5,8,9^* alleles, pML468.6 and pML488.15, were kind gifts of Maria Pia Longhese (Università degli Studi di Milano-Bicocca). The *SAE2*-bearing fragments from pML468.6 and pML488.15 were subcloned into the *Eco*RI site of pRS313 and verified by sequencing to generate pRDK1698 and pRDK1699. The wild-type *SAE2*-bearing plasmid, pRDK1700, was generated by PCR amplification of *SAE2* from wild-type genomic DNA with primers 5′-TGC AAT AGA GTC GTG AAT TCG TCT GAG TTA GCG TCT GAT TTT GAC TCT TTC TTC TTC TTT TTC GTC TT-3′ and TGC AAT AGA GTC GTG AAT TCC CTG GTA GTT AGG TGT CAT TTG TTT AAC GTC CGT TAA CTT CCC CTT TCT-3′ to generate an insert spanning the same genomic region as pRDK1698 and pRDK1699. The *sae2-S267A* plasmid, pRDK1701, was generated by site-directed mutagenesis of pRDK1700 and verified by sequencing. For GCR rate determination, the transformed query strains were grown in –HIS liquid media, viable cell determination was performed by plating on –HIS media, and GCR-containing progeny were selected on Can/5-FOA media lacking histidine.

Alleles of *mre11* were introduced into the *mre11::HIS3* deletion strains using pRS314 *ARS CEN* plasmids containing the *TRP1* marker. The wild-type *MRE11* plasmid was generated by PCR amplifying *MRE11* from wild-type genomic DNA with the primers 5′-CTG AGG AAT TCG ATT TGG CTA AAC TAG GCT GAG GTA GGC TCG-3′ and 5′-CTG AGC TCG AGG GTA TTG TTT CCC ACA AGG GGA CGG TTA ATG-3′ and cloning the PCR product into pRS314 cut with *Eco*RI and *Xho*I. The resulting plasmid, pRDK1702, was verified by sequencing. The *mre11-H125N* and *mre11S* (*mre11-P84S,T188I*) plasmids, pRDK1703 and pRDK1704, were generated by site-directed mutagenesis and verified by sequencing. The *mre11-2* and *mre11-3* plasmids, pRS314-mre11-2 and pRS314-mre11-3, were kind gifts of John Petrini (Sloan-Kettering Institute). For GCR rate determination, the transformed query strains were grown in –TRP liquid media, viable cell determination was performed by plating on –TRP media, and GCR-containing progeny were selected on GCR media lacking tryptophan.

### Determination of GCR rates and *hph* retention

GCR rates were determined using multiple independent biological isolates as previously described [71]. The frequency of *hph* retention was determined by testing a single GCR-containing isolate from each of a number of individual independent cultures for growth on YPD media supplemented with 200 µg/mL hygromycin B (Invitrogen).

### Statistical analysis

The significance of the deviation of *hph*-retention for each genotype was measured using the maximum likelihood statistical significance G-test [72] as implemented for R by P. Hurd (http://www.psych.ualberta.ca/~phurd/cruft/). Probabilities for the null model that the observed distributions were generated by the same underlying rate were calculated using the two-tailed Mann-Whitney U-test (http://faculty.vassar.edu/~lowry/utest.html). A significant differences was inferred when the probability of the null model was 0.01 or less.

### PFGE gel and Southern blotting

DNA plugs for PFGE were prepared as described [73]. *Asc* I-digested plugs were prepared by treating plugs with 50 units of *Asc* I (New England Biolabs) overnight at 37°C. Electrophoresis was performed using a Bio-Rad CHEF-DRII apparatus at 7 V/cm, with a 60 to 120 s switch time for 24 h. The gels were stained with ethidium bromide and imaged. The DNA in the gel was transferred to Hybond-XL membranes by neutral capillary blotting. The DNA was crosslinked to the membrane by UV irradiation in a Stratalinker™ (Stratagene) apparatus at maximum output for 60 seconds. Probes were generated by random primer labeling of *MCM3* and *hph* fragments with the Prime-It II kit (Stratagene). Probe hybridization was performed at 68°C for 2–4 hr. The membrane was then washed extensively and imaged with a PhosphoImager (Molecular Dynamics, Inc.).

### Multiplex ligation-mediated probe amplification analysis

Primers targeted to the left arm of chromosome V (Figure S7A) were designed according to the recommendations on the MRC-Holland website (http://www.mrc-holland.com) with the length of each amplification product differing by 6 basepairs (Table S4). The reagents were purchased from MRC-Holland, and the amplification, fragment separation, and fragment detection steps were performed essentially as described [28]. Data were collected on an ABI 3730XL sequencer using the POP7 polymer and GS500-LIZ sizing standard (Life Technologies). The raw data for each run were integrated using GeneMapper software (Life Technologies) and analyzed using a custom Python script that uses gnuplot (http://www.gnuplot.info) to plot the integrated area for each peak in wild-type controls against the respective peak in experimental samples (Figure S7B and C). Amplification detected by MLPA was verified by comparison with aCGH data for isolates 213, 217, 362, and 3178 (Figure S7D).

### Array comparative genomic hybridization

One µg of genomic DNA was prepared from GCR-containing isolates and the wild-type strain RDKY6677 using the Purgene kit (Qiagen) and concentrated to >100 ng µL^−1^. The DNA from GCR-containing isolates was amplified and labeled with Cy5, and wild-type control DNA was amplified and labeled with Cy3. Subsequently, four mixtures containing GCR isolate/wild-type pairs were hybridized to a NimbleGen 4-plex chip. Data were analyzed using the SignalMap software (NimbleGen) and remapped from the chrV sequence of the reference genome to the coordinates of chrV in RDKY6677. Microarray data have been deposited at ArrayExpress (http://www.ebi.ac.uk/arrayexpress) with under the accession E-MTAB-2377.

### Whole genome paired-end sequencing

Multiplexed paired-end libraries were constructed from 5 µg of genomic DNA purified using the Purgene kit (Qiagen). The genomic DNA was sheared by sonication and end-repaired using the End-it DNA End-repair kit (Epicentre Technologies). Common adaptors from the Multiplexing Sample Preparation Oligo Kit (Illumina) were then ligated to the genomic DNA fragments, and the fragments were then subjected to 18 cycles of amplification using the Library Amplification Readymix (KAPA Biosystems). The amplified products were fractionated on an agarose gel to select 600 bp fragments, which were subsequently sequenced on an Illumina HiSeq 2000 using the Illumina GAII sequencing procedure for paired-end short read sequencing. Reads from each read pair were mapped separately by bowtie version 0.12.7 [74] to a reference sequence that contained revision 64 of the *S. cerevisiae* S288c genome (http://www.yeastgenome.org), *hisG* from *Samonella enterica*, and the *hphMX4* marker (Table S1). Sequencing data have been deposited at NCBI Sequence Read Archive (http://www.ncbi.nlm.nih.gov/sra) under the accession SRP039033.

### Rearrangement and copy number analysis of paired-end sequencing data

Chromosomal rearrangements were identified after bowtie mapping by version 0.5 of the Pyrus suite (http://www.sourceforge.net/p/pyrus-seq). Briefly, after PCR removal of PCR duplicates, read pairs with 2 uniquely mapping reads were used to generate 2 distributions. The number of times each base pair was read (the ‘nread’ distribution) was determined for identifying the sequence variants observed a significant number of times, and the number of times each base pair was spanned by a pair of reads (the ‘nspan’ distribution) was determined to identify the candidate chromosomal rearrangements that were supported by a significant number of read pairs. The data were then analyzed for junction-defining read pairs that indicated the presence of structural rearrangements relative to the reference genome, such as the *tel1::HIS3* deletion or GCR-related fold-back inversions. The junction-sequencing reads were identified from read pairs in which one read could not be mapped and the other read mapped next to the junction-defining read pairs. Sequences of the junctions were generated by *de novo* alignment of the junction-sequencing reads associated with rearrangements defined by statistically significant junction-defining read pairs (Figure S3). The identified rearrangements included all known rearrangements in the strains that could be defined based on the average distance between the read pairs in the library (Table S2).

## Supporting Information

Figure S1 PFGE analysis of *hph*+ GCR-containing isolates from the *tel1Δ* uGCR assay strain. (**A and C**) Southern blot using an *hph* probe of a pulsed-field gel (PFG) of the wild-type strain (RDKY6677) and 6 GCR-containing isolates with and without *Asc*I treatment. (**B and D**) Southern blot of a second PFG with identical samples as in panel A or C using a *MCM3* probe.
(PDF)

Figure S2 Analysis of interstitial deletions from the *tel1Δ* uGCR assay strain. Sequence of the junction (middle line) is displayed between the sequences of the two target regions. Bases between colons are identical in both joined fragments. Coordinates of the breakpoint mapped to the two targets are reported above and below the alignment for both the reference S288c genome sequence and the uGCR chrV. For isolate 214, the fusion is to a Ty element. For the 214 “left” junction, the Ty elements in the reference genome that best matched the junction sequence were *YERCTy1-1*, *YMLWTy1-2*, *YPLWTy1-1*, *YGRWTy1-1*, *YDRWTy1-5*, *YOLWTy1-1*, *YLRWTy1-2*, *YLRWTy1-3*, *YLRCTy1-1*, *YLRWTy1-1*, and *YPRCTy1-4*. For the 214 right junction, the Ty-related junction sequence mapped to a large number of Ty-related elements.
(PDF)

Figure S3 Searching and subsequent identification of rearrangements by the Pyrus programs. (**A**) For a novel rearrangement, depicted here as a translocation between chrA (white) and chrB (gray), junction-defining read pairs are read pairs for which both read pairs map (black arrows separated by a dashed line): one read pair maps to chrA and one read pair maps to chrB. To defined as belonging to the same rearrangement ‘event’, reads mapped to each target must additionally (i) have the same orientation as the other reads that map to that target and (ii) map within a short distance (defined based on the distribution of distances between read pairs) of other read pairs indicating the same event. Importantly, because junction-defining read pairs map to each target, these read pairs must span any novel junction and, in general, cannot sequence the junction. Junction sequencing reads, however, can be identified as non-mapping (red arrows) reads associated in read pairs with other reads that map uniquely to the two targets in the vicinity of junction-defining read pairs. Alignment of junction sequencing reads can identify the sequence. (**B**) Mapping of the 572 junction-defining read pairs and 114 junction-sequencing read pairs for the chrV interstitial deletion in isolate 3118. Junction-defining read pairs are sorted by the mapped position of telomeric marker. The position of the junction-sequencing reads (red arrows) is arbitrary. Note that the reads paired with the junction sequencing reads are in the vicinity and have the same orientation as the junction-defining read pairs. (**C**) The junction sequence derived from alignment of the junction-sequencing reads for the isolate 3118 interstitial deletion is displayed on the second line. The telomeric sequence alignment is on the top line and the centromeric alignment is on the bottom line. Bases of identity between the two targets are surrounded by colons. (**D**) The interstitial deletion junction sequence derived by PCR amplification and Sanger sequencing for isolate 3118 is identical to the sequence derived by alignment of the junction-sequencing reads.
(PDF)

Figure S4 Examples of PCR mapping indicating the presence of a *URA3/ura3-52* fusion junction. (**A**) A primer located telomeric to the *yel068c::CAN1/URA3* insertion (fore) and a primer within the 3′ end of *URA3* (rev1) amplify an identical fragment in the starting and GCR-containing strains. (**B**) The fore primer and a primer within the end of Ty element (rev2) only amplifies products in strains with a *URA3/ura3-52* junction. (**C**) The fore primer and a primer within the 5′ end of *URA3* (rev3) amplify a ∼1.8 kb fragment in the starting strain, but a large, ∼8 kb fragment in strains with a *URA3/ura3-52* junction, consistent with the presence of a Ty element. (**D**) The fore primer and a primer telomeric to *ura3-52* (rev4) only amplifies a large ∼8 kb fragment in strains with a *URA3/ura3-52* junction.
(PDF)

Figure S5 Formation and sequences of the inversion junctions in the sequenced inverted duplication GCRs. Predicted mechanism of hairpin formation and the sequence of the inversion junction for the inverted duplication class of GCRs. The arrow indicates the position of the double strand break (DSB) under the simplest scenario in which the 3′ end of the DSB is the 3′ end used to initiate the inversion; however, more complicated scenarios with more distal initiating DSBs have been observed [75]. 5′->3′ resection generates a single-stranded region that can mediate the formation of a single-stranded hairpin that can anneal and extend from the exposed single stranded region. Regions of homology involved in annealing are underlined. Duplicated palindromic sequence in the replicated product and the annotated junction sequence are displayed with arrows. Sequences derived from alignment of the junction sequencing reads are displayed at bottom. Panel **A** depicts the inversion junction for isolate 217; panel **B** depicts the inversion junction for isolate 218, 362, 364, and 366; panel **C** depicts the inversion junction for isolate 365; panel **D** depicts the inversion junction for isolate 2977; panel **E** depicts the inversion junction for isolate 3124; panel **F** depicts the inversion junction for isolates 3121 and 3125; and panel **G** depicts the inversion junction for isolate 3255.
(PDF)

Figure S6 Analysis of *hph*− GCR-containing isolates from the *tel1Δ* uGCR assay strain. (**A**) Southern blot using a *MCM3* probe of a PFG of the wild-type strain (RDKY6677) and 13 *hph−* GCR isolates revealed that isolates 213, 2976, 3124, and 3125 had a rearranged chrV that was substantially larger than wild-type, whereas the other isolates had rearranged chrV that was similar to wild-type. (**B**) Sequences of some of the breakpoints from GCRs associated with a normal-sized chrV showed the GCRs involved translocations (isolate 213) or *de novo* telomere additions (isolates 3116, 3117, and 3120). For isolate 216, the junction sequence is displayed as in Figure S2 and the Ty elements in the reference genome that best matched the junction sequence were *YJRWTy1-2* and *YGRWTy1-1*.
(PDF)

Figure S7 Example of the multiplex ligation-mediated probe amplification (MLPA) analysis used to identify copy number changes along the left arm of chrV. (**A**) Diagram illustrating the position of the MLPA probes relative to Ty-related sequences (open boxes) on chrVL. (**B**) MLPA analysis of isolate 362, which had a larger-than-wild-type rearranged chrV (Fig. S1), revealed that the peak areas corresponding to probes in the genes *PCM1*, *VMA8*, *BUD16*, and *GEA2* (boxed labels) were amplified relative to the peak areas from the wild-type strain, whereas probes in the genes *EAF5*, *YEA6*, *IRC22*, and *MNN1* were not. This pattern of amplification is consistent with a duplication spanning a region telomeric to *PCM1* until *ura3-52*. (**C**) MLPA analysis of isolate 3175 revealed that there was no change in copy number on chrV relative to the wild-type strain. (**D**) Mapping the amplified MLPA probes to the aCGH data for isolate 362 revealed that MLPA and aCGH yielded consistent results on the extent of the chrV amplification (compare the boxed MLPA probes with the red line indicating duplication in the aCGH data).
(PDF)

Figure S8 Analysis of *hph*+ GCR-containing isolates from the wild-type uGCR assay strain. (**A**) Southern blot using an *hph* probe of a PFG of the wild-type strain (RDKY6677), isolate 3178, and isolate 3255 with and without *Asc*I treatment. **B.** Southern blot of a second PFG with identical samples as in panel A using an *MCM3* probe. **C.** The log base 2 ratio of the aCGH hybridization intensity on chrVL for isolate 3255. The solid horizontal bar is at 0 and dashed lines are at −1 and 1 (2-fold decreased and increased, respectively). Probes were mapped onto the “uGCR Chromosome V” coordinate system. Chromosomal features including *hph*, the *CAN1/URA3* cassette, the *ura3-52* mutation, and the centromere (*CEN5*) are indicated at top. Red bracket displays the duplicated chromosomal region. **D.** Breakpoint sequence of the interstitial deletion in the *CAN1/URA3* cassette from isolate 3255 is displayed in the center line aligned with homologies to *URA3* (top line) and *CAN1* (bottom line). Sequence between colons indicates the homology at the breakpoint junction. Coordinates of the sequences are given relative to the uGCR chrV.
(PDF)

Figure S9 Potential mechanisms for initiation of BIR by hairpin-capped DSBs and invasion of the telomeric *hph*-containing chrV fragment by BIR products. (**A**) After formation of the hairpin-capped DSB (Fig. S5), the 3′ end of the hairpin (black arrow) can be used to drive BIR similarly to an invading strand from another duplex. Replication can proceed either by a migrating D-loop mechanism that transiently displaces the complementary strand (grey) or a mechanism in which a new replication fork is established. The three grey arrows indicate lagging strand replication. (**B**) The mechanisms for capture of the telomeric *hph*-containing fragment of chrV described in the discussion are illustrated. In mechanism 1 (BIR and template switching), extension from the hairpin terminates before the entire chrV is copied, and the dissociated 3′ end invades the *hph*-containing fragment via intermolecular BIR mediated by the homology between *ura3-52* and *URA3*. In mechanism 2 (BIR and isochromosome formation), BIR copies the entire chrV, generating a dicentric isochromosome that breaks during replication and then captures the *hph*-containing fragment by intermolecular BIR. In mechanism 3 (covalent closure and isochromosome formation), the hairpin is extended and then ligated to the complementary strand. Replication of this molecule generates a dicentric isochromosome that then breaks and captures the *hph*-containing fragment as in mechanism 2.
(PDF)

Table S1 Statistics for Next Generation Sequencing results.
(PDF)

Table S2 Number of junction-defining read pairs and junction-sequencing reads for isolates with sequenced genomes.
(PDF)

Table S3 *S. cerevisiae* strains.
(PDF)

Table S4 MLPA primers.
(PDF)
