# Supplementary figures and images for: DNA Repair Pathway Selection Caused by Defects in TEL1, SAE2, and De Novo Telomere Addition Generates Specific Chromosomal Rearrangement Signatures
Source: PLoS Genet. 2014 Apr 3;10(4):e1004277. doi: 10.1371/journal.pgen.1004277 (PMC3974649; doi:10.1371/journal.pgen.1004277)

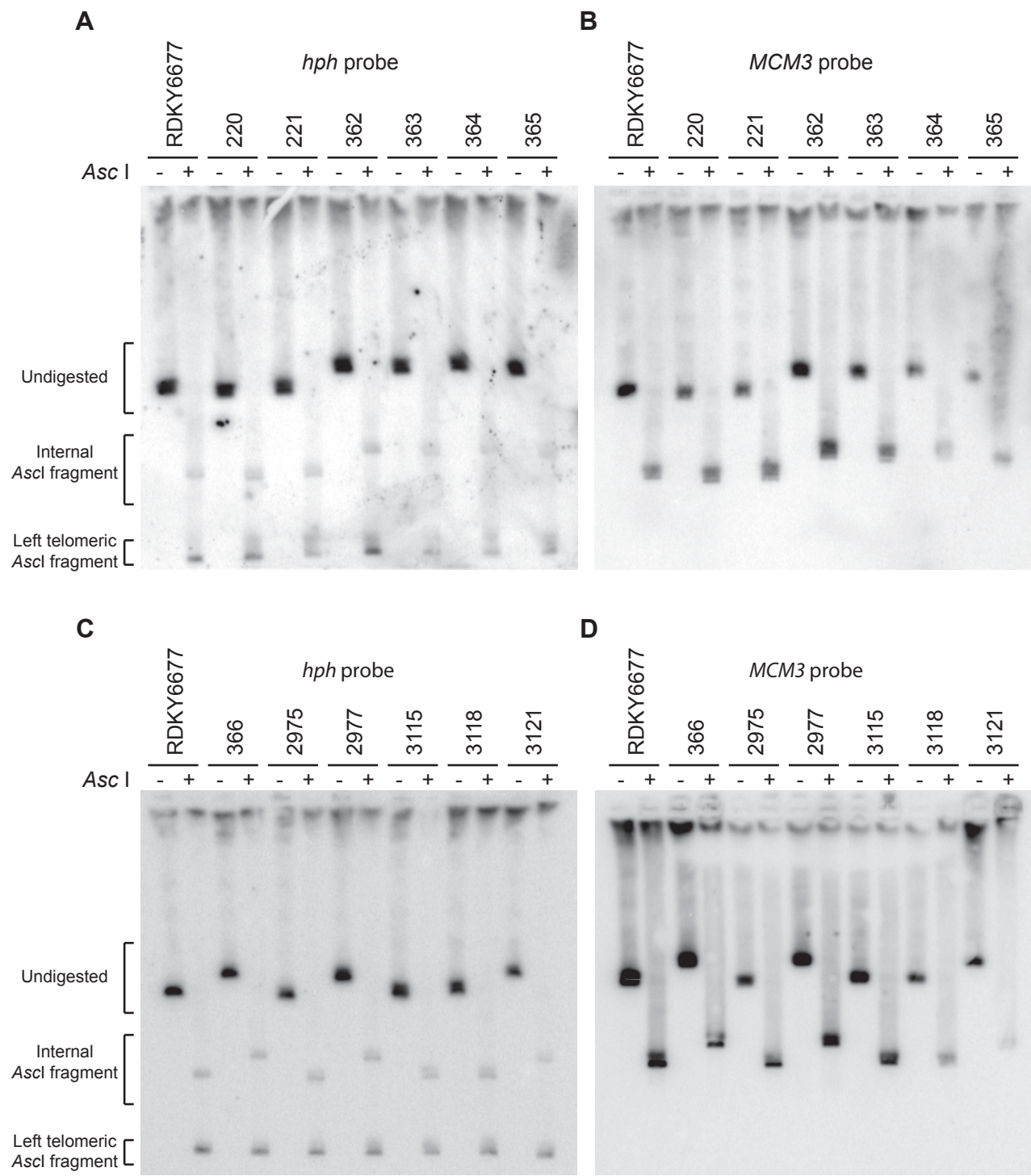

Supplement: Figure S1 — PFGE analysis of hph+ GCR-containing isolates from the tel1Δ uGCR assay strain. (A and C) Southern blot using an hph probe of a pulsed-field gel (PFG) of the wild-type strain (RDKY6677) and 6 GCR-containing isolates with and without AscI treatment. (B and D) Southern blot of a second PFG with identical samples as in panel A or C using a MCM3 probe. (PDF) [file pgen.1004277.s001.pdf]

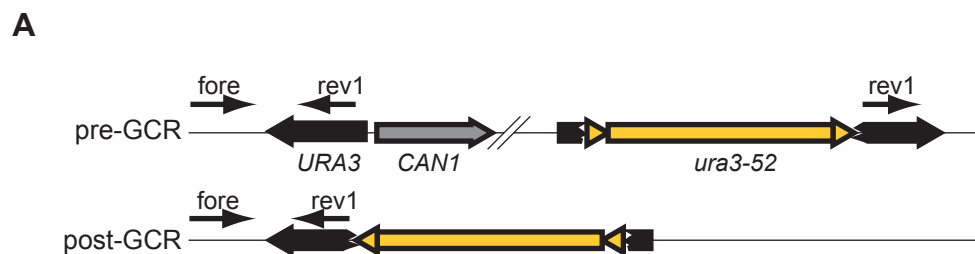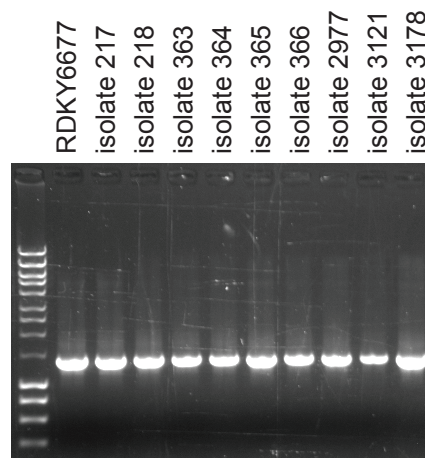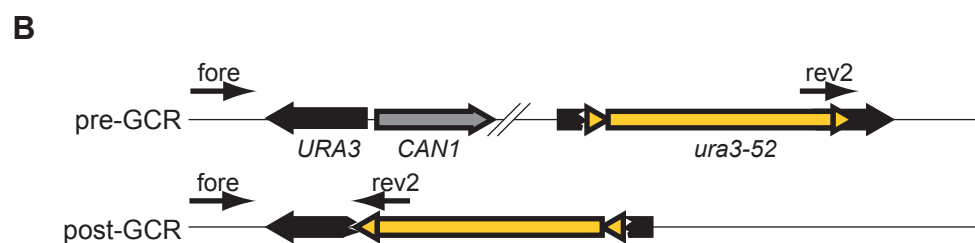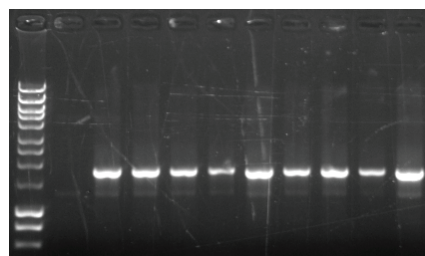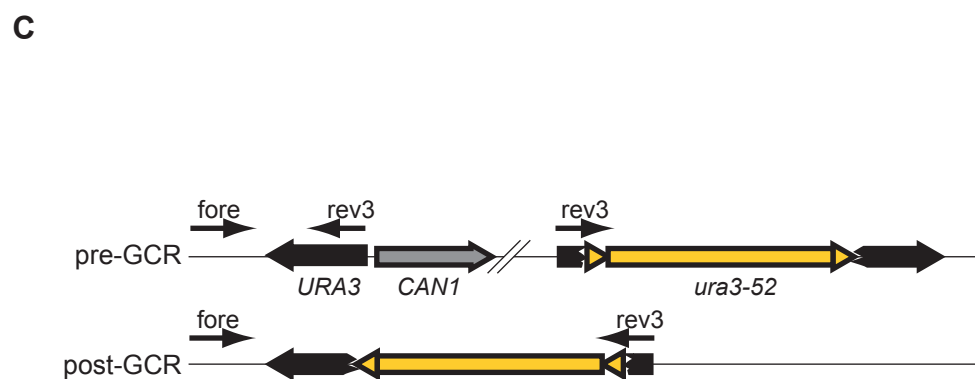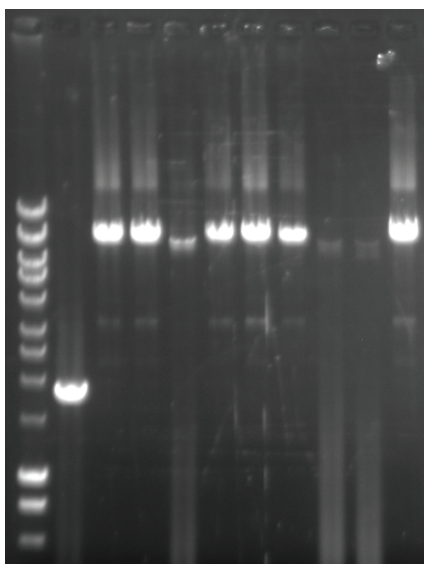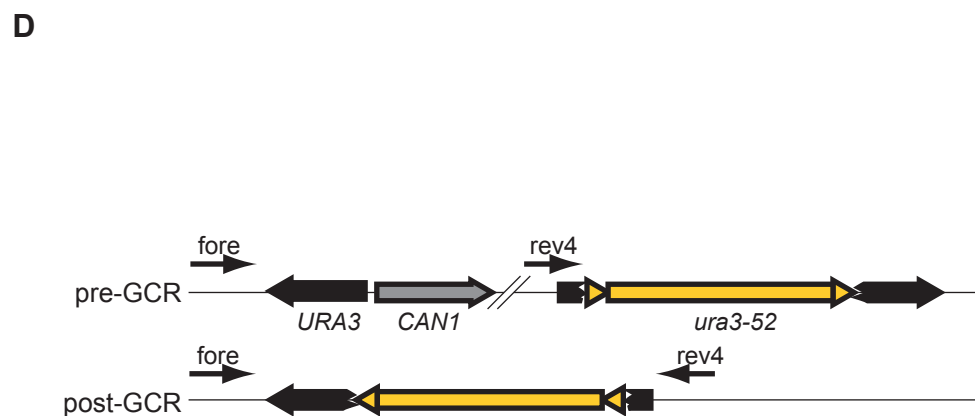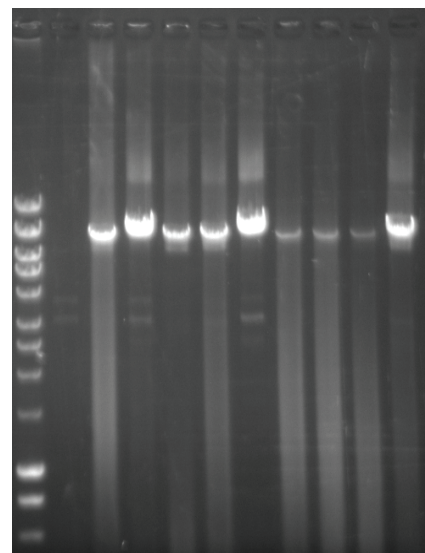

Supplement: Figure S4 — Examples of PCR mapping indicating the presence of a URA3/ura3-52 fusion junction. (A) A primer located telomeric to the yel068c::CAN1/URA3 insertion (fore) and a primer within the 3′ end of URA3 (rev1) amplify an identical fragment in the starting and GCR-containing strains. (B) The fore primer and a primer within the end of Ty element (rev2) only amplifies products in strains with a URA3/ura3-52 junction. (C) The fore primer and a primer within the 5′ end of URA3 (rev3) amplify a ∼1.8 kb fragment in the starting strain, but a large, ∼8 kb fragment in strains with a URA3/ura3-52 junction, consistent with the presence of a Ty element. (D) The fore primer and a primer telomeric to ura3-52 (rev4) only amplifies a large ∼8 kb fragment in strains with a URA3/ura3-52 junction. (PDF) [file pgen.1004277.s004.pdf]

Putnam et al. 2014. Figure S7.

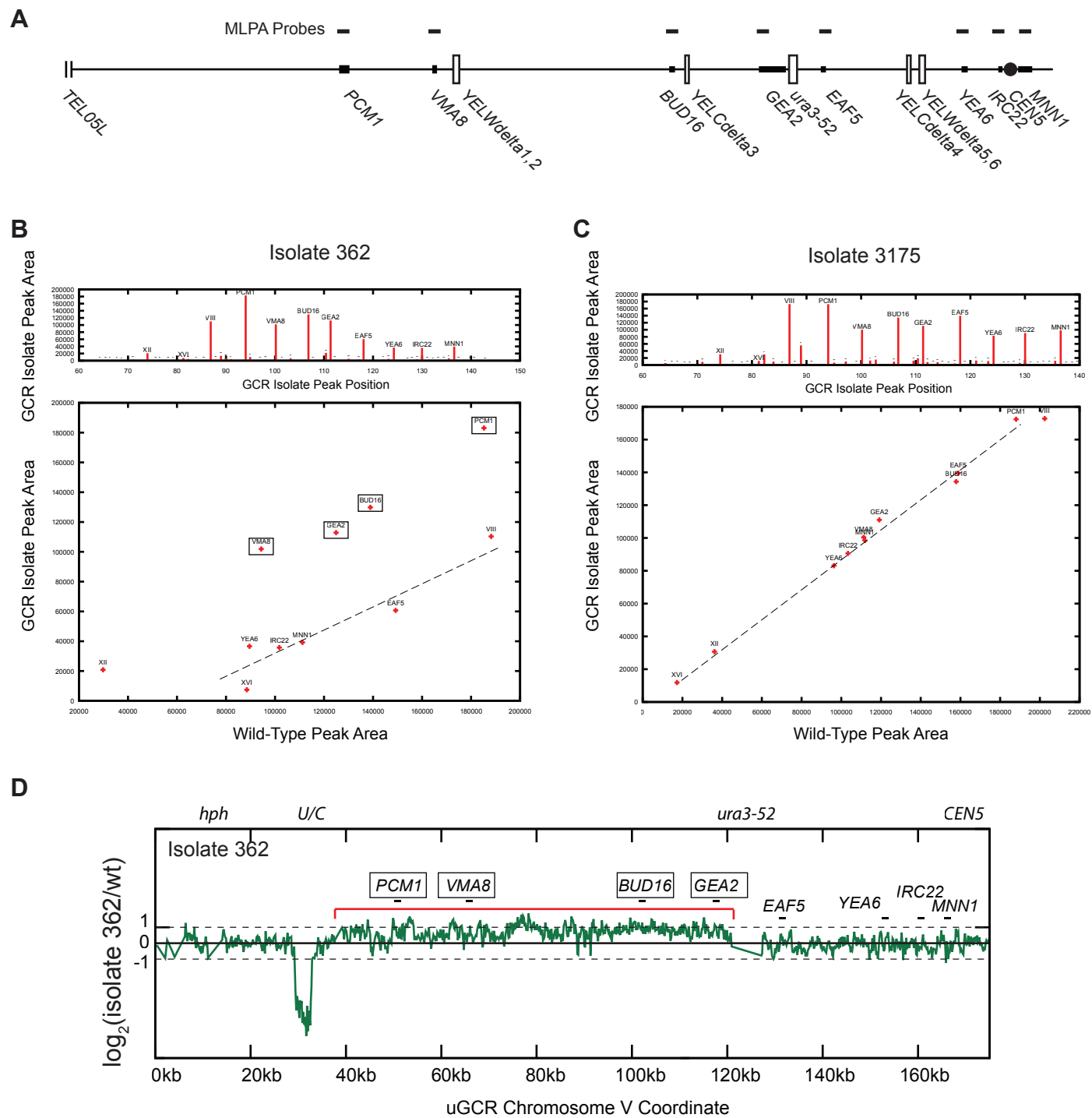

Supplement: Figure S7 — Example of the multiplex ligation-mediated probe amplification (MLPA) analysis used to identify copy number changes along the left arm of chrV. (A) Diagram illustrating the position of the MLPA probes relative to Ty-related sequences (open boxes) on chrVL. (B) MLPA analysis of isolate 362, which had a larger-than-wild-type rearranged chrV (Fig. S1), revealed that the peak areas corresponding to probes in the genes PCM1, VMA8, BUD16, and GEA2 (boxed labels) were amplified relative to the peak areas from the wild-type strain, whereas probes in the genes EAF5, YEA6, IRC22, and MNN1 were not. This pattern of amplification is consistent with a duplication spanning a region telomeric to PCM1 until ura3-52. (C) MLPA analysis of isolate 3175 revealed that there was no change in copy number on chrV relative to the wild-type strain. (D) Mapping the amplified MLPA probes to the aCGH data for isolate 362 revealed that MLPA and aCGH yielded consistent results on the extent of the chrV amplification (compare the boxed MLPA probes with the red line indicating duplication in the aCGH data). (PDF) [file pgen.1004277.s007.pdf]

Putnam et al. 2014. Figure S8.

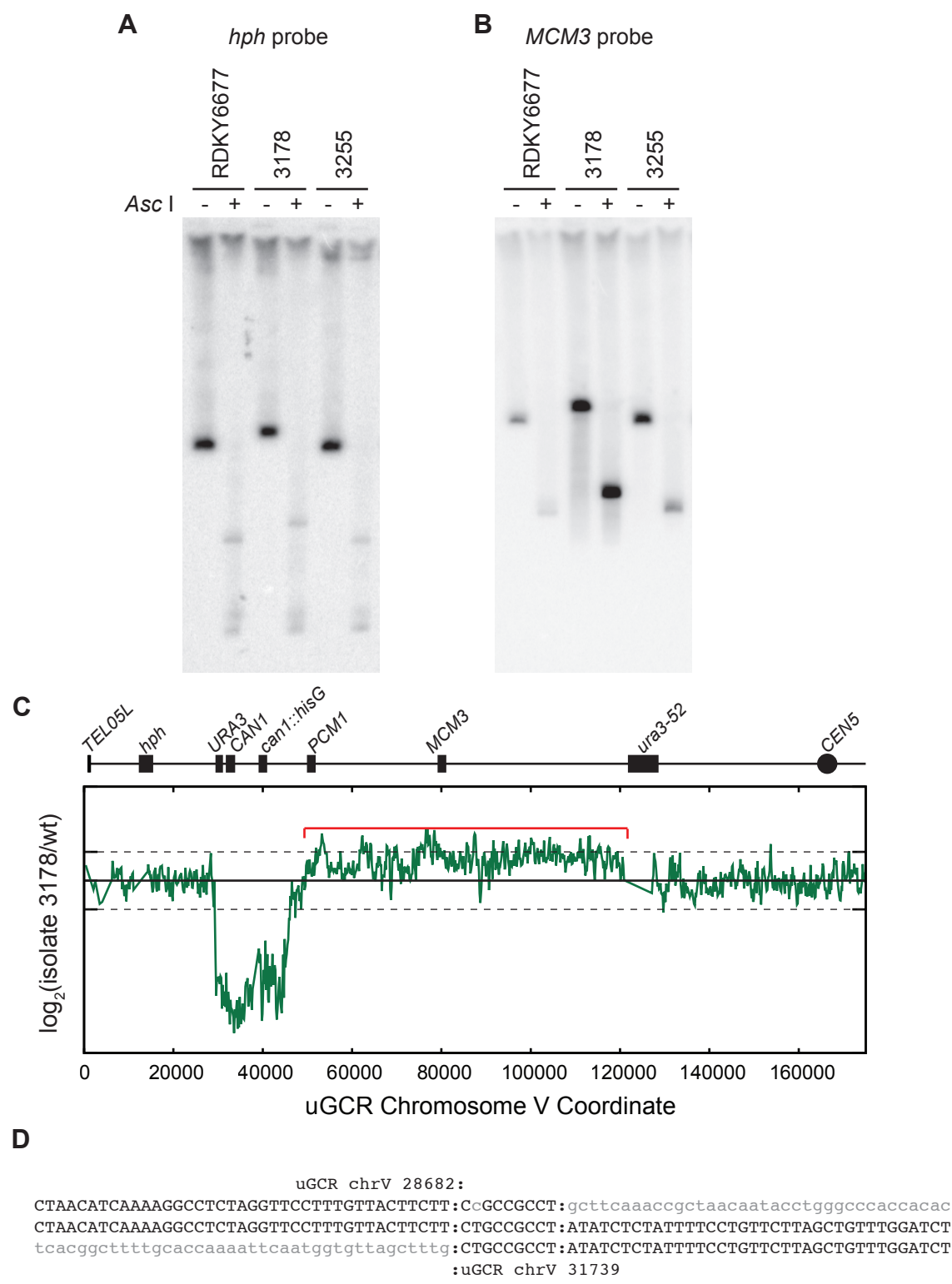

Supplement: Figure S8 — Analysis of hph+ GCR-containing isolates from the wild-type uGCR assay strain. (A) Southern blot using an hph probe of a PFG of the wild-type strain (RDKY6677), isolate 3178, and isolate 3255 with and without AscI treatment. B. Southern blot of a second PFG with identical samples as in panel A using an MCM3 probe. C. The log base 2 ratio of the aCGH hybridization intensity on chrVL for isolate 3255. The solid horizontal bar is at 0 and dashed lines are at −1 and 1 (2-fold decreased and increased, respectively). Probes were mapped onto the “uGCR Chromosome V” coordinate system. Chromosomal features including hph, the CAN1/URA3 cassette, the ura3-52 mutation, and the centromere (CEN5) are indicated at top. Red bracket displays the duplicated chromosomal region. D. Breakpoint sequence of the interstitial deletion in the CAN1/URA3 cassette from isolate 3255 is displayed in the center line aligned with homologies to URA3 (top line) and CAN1 (bottom line). Sequence between colons indicates the homology at the breakpoint junction. Coordinates of the sequences are given relative to the uGCR chrV. (PDF) [file pgen.1004277.s008.pdf]

A.

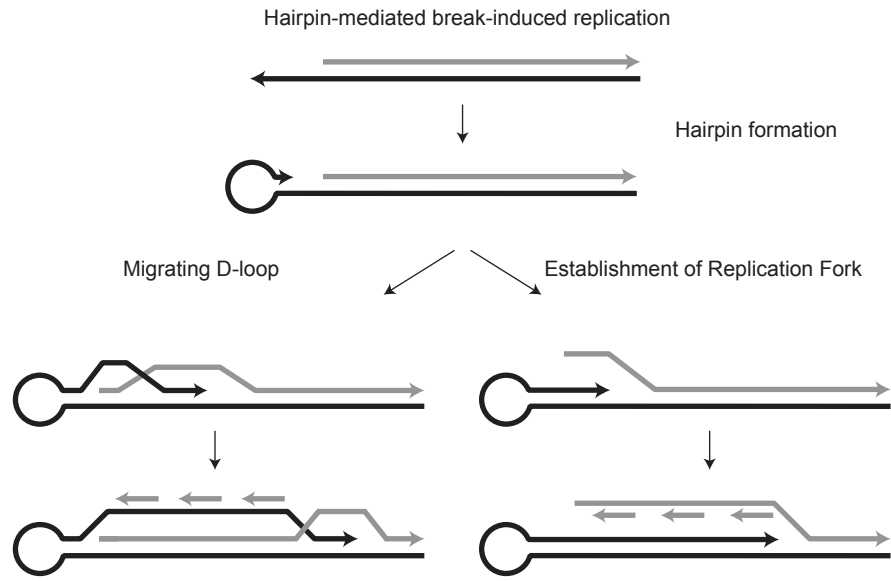

B.

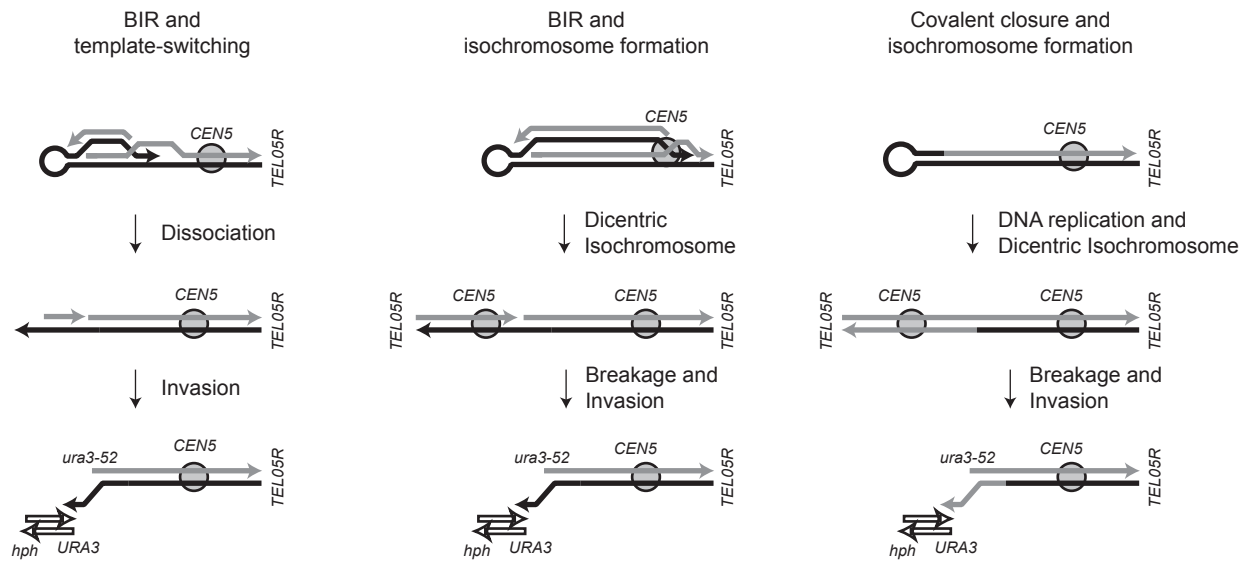

Supplement: Figure S9 — Potential mechanisms for initiation of BIR by hairpin-capped DSBs and invasion of the telomeric hph-containing chrV fragment by BIR products. (A) After formation of the hairpin-capped DSB (Fig. S5), the 3′ end of the hairpin (black arrow) can be used to drive BIR similarly to an invading strand from another duplex. Replication can proceed either by a migrating D-loop mechanism that transiently displaces the complementary strand (grey) or a mechanism in which a new replication fork is established. The three grey arrows indicate lagging strand replication. (B) The mechanisms for capture of the telomeric hph-containing fragment of chrV described in the discussion are illustrated. In mechanism 1 (BIR and template switching), extension from the hairpin terminates before the entire chrV is copied, and the dissociated 3′ end invades the hph-containing fragment via intermolecular BIR mediated by the homology between ura3-52 and URA3. In mechanism 2 (BIR and isochromosome formation), BIR copies the entire chrV, generating a dicentric isochromosome that breaks during replication and then captures the hph-containing fragment by intermolecular BIR. In mechanism 3 (covalent closure and isochromosome formation), the hairpin is extended and then ligated to the complementary strand. Replication of this molecule generates a dicentric isochromosome that then breaks and captures the hph-containing fragment as in mechanism 2. (PDF) [file pgen.1004277.s009.pdf]
