# Supplementary material for: DNA Repair Pathway Selection Caused by Defects in TEL1, SAE2, and De Novo Telomere Addition Generates Specific Chromosomal Rearrangement Signatures
Source: PLoS Genet. 2014 Apr 3;10(4):e1004277. doi: 10.1371/journal.pgen.1004277 (PMC3974649; doi:10.1371/journal.pgen.1004277)
Supplement: Figure S2 — Analysis of interstitial deletions from the tel1Δ uGCR assay strain. Sequence of the junction (middle line) is displayed between the sequences of the two target regions. Bases between colons are identical in both joined fragments. Coordinates of the breakpoint mapped to the two targets are reported above and below the alignment for both the reference S288c genome sequence and the uGCR chrV. For isolate 214, the fusion is to a Ty element. For the 214 “left” junction, the Ty elements in the reference genome that best matched the junction sequence were YERCTy1-1, YMLWTy1-2, YPLWTy1-1, YGRWTy1-1, YDRWTy1-5, YOLWTy1-1, YLRWTy1-2, YLRWTy1-3, YLRCTy1-1, YLRWTy1-1, and YPRCTy1-4. For the 214 right junction, the Ty-related junction sequence mapped to a large number of Ty-related elements. (PDF) [file pgen.1004277.s002.pdf]

Putnam et al. 2014. Figure S2.

Isolate | Junction Sequence

212

```
 URA3 insertion:
uGCR ChrV 28999:
 CAATGAAGCACACA::AGTTTGTTTGCTTTTTC
 CAATGAAGCACACA::CATAACATTTGGTATAA
 AGTCAAGAACATAT::CATAACATTTGGTATAA
                :ChrV 29606
                :uGCR ChrV 36496
```

214 (left)

```
         ChrV 12870:
    uGCR ChrV 15213:
TTTGGTGAGATAGTTT:ACG:CCTATAATATATA
TTTGGTGAGATAGTTT:ACG:TCATCGACATCTA
                 :ACG:TCATCGACATCTA
                 :Ty element
```

214 (right)

```
         Ty element:
TGGGTGGTATGTTGGAA:TA:
TGGGTGGTATGTTGGAA:TA:TTAGGAATGCCG
GGTATTCTTGAAGAGTC:TA:TTAGGAATGCCG
                    :ChrV 36143
                    :uGCR ChrV 40936
```

219

```
         ChrV 16051:
    uGCR ChrV 18395:
GCAATGTCAACAGGAG:TG:TCGCATTTTATGTTTTTT
GCAATGTCAACAGGAG:TG:TGCTATATAGAGTATATG
TTATTTATCACACTAC:TG:TGCTATATAGAGTATATG
                   :ChrV 42374
                   :uGCR ChrV 47186
```

220

```
        ChrV 25459:
   uGCR ChrV 27802:
TCAAATTGGAAG:TAAAT:AAATAAGGACTG
TCAAATTGGAAG:TAAAT:CGGTCTTTGATC
TTGATCAAAGAT:TAAAT:CGGTCTTTGATC
                  :ChrV 40094
                  :uGCR ChrV 44887
```

Isolate | Junction Sequence

221

```
        ChrV 19099:
   uGCR ChrV 21442:
ACTCTCTCCTTGGCA::CGGACCTAAAAATAAC
ACTCTCTCCTTGGCA::GAGTATTCTAAATTAG
ATGTCTGAGTTAGGT::GAGTATTCTAAATTAG
                :CAN1 insertion
                :uGCR ChrV 32399
```

2975

```
            ChrV 16981:
       uGCR ChrV 19324:
GATTATCAGCAATATCAAT:GCC:TAAGGAAGGACAATACT
GATTATCAGCAATATCAAT:GCC:TGCAGTGTACTGGGTTA
GTGCCGATGAGCTCGGTAC:GCC:TGCAGTGTACTGGGTTA
                       :ChrV 27098
                       :uGCR ChrV 33988
```

3115

```
           ChrV 25355:
      uGCR ChrV 27698:
ATTATGACATTTGCAGGACT::CACACCCAGAAGTCCTTTC
ATTATGACATTTGCAGGACT::GCTGGAAGATCTGAATTCT
TCTCATGCGTTCATGCACCA::GCTGGAAGATCTGAATTCT
                     :can1::hisG insertion
```

3118

```
           ChrV 24590:
      uGCR ChrV 26930:
AAATTATTATCATTATC:GCT:ACTTCAGCGATTTCAATTT
AAATTATTATCATTATC:GCT:TTGCATCTGATGCTGGCTC
ATATCAGTCTTTGCATG:GCT:TTGCATCTGATGCTGGCTC
                     :ChrV 29648
                     :uGCR ChrV 36538
```
