# Supplementary material for: DNA Repair Pathway Selection Caused by Defects in TEL1, SAE2, and De Novo Telomere Addition Generates Specific Chromosomal Rearrangement Signatures
Source: PLoS Genet. 2014 Apr 3;10(4):e1004277. doi: 10.1371/journal.pgen.1004277 (PMC3974649; doi:10.1371/journal.pgen.1004277)
Supplement: Figure S3 — Searching and subsequent identification of rearrangements by the Pyrus programs. (A) For a novel rearrangement, depicted here as a translocation between chrA (white) and chrB (gray), junction-defining read pairs are read pairs for which both read pairs map (black arrows separated by a dashed line): one read pair maps to chrA and one read pair maps to chrB. To defined as belonging to the same rearrangement ‘event’, reads mapped to each target must additionally (i) have the same orientation as the other reads that map to that target and (ii) map within a short distance (defined based on the distribution of distances between read pairs) of other read pairs indicating the same event. Importantly, because junction-defining read pairs map to each target, these read pairs must span any novel junction and, in general, cannot sequence the junction. Junction sequencing reads, however, can be identified as non-mapping (red arrows) reads associated in read pairs with other reads that map uniquely to the two targets in the vicinity of junction-defining read pairs. Alignment of junction sequencing reads can identify the sequence. (B) Mapping of the 572 junction-defining read pairs and 114 junction-sequencing read pairs for the chrV interstitial deletion in isolate 3118. Junction-defining read pairs are sorted by the mapped position of telomeric marker. The position of the junction-sequencing reads (red arrows) is arbitrary. Note that the reads paired with the junction sequencing reads are in the vicinity and have the same orientation as the junction-defining read pairs. (C) The junction sequence derived from alignment of the junction-sequencing reads for the isolate 3118 interstitial deletion is displayed on the second line. The telomeric sequence alignment is on the top line and the centromeric alignment is on the bottom line. Bases of identity between the two targets are surrounded by colons. (D) The interstitial deletion junction sequence derived by PCR amplification and Sange [file pgen.1004277.s003.pdf]

Putnam et al. 2014. Figure S3.

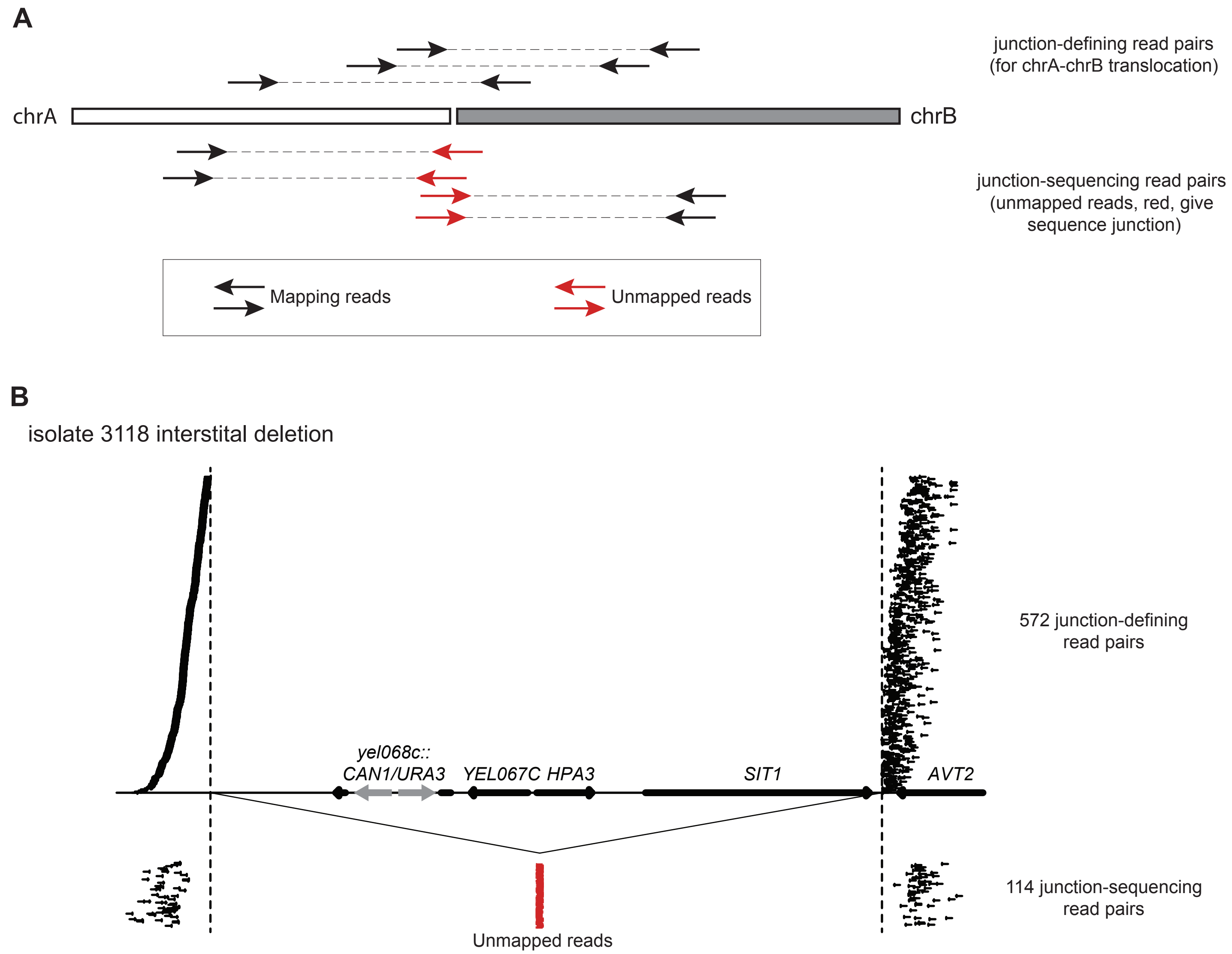

C

```
                                                  ChrV 24590:
ATTGCGGAAGGAGCTTTGCTCTGGTTGAAATTATTATCATTATC:GCT:
ATTGCGGAAGGAGCTTTGCTCTGGTTGAAATTATTATCATTATC:GCT:TTGCATCTGATGCTGGCTCTACCGACTTCTCGGCGATAAAAT
                                            :GCT:TTGCATCTGATGCTGGCTCTACCGACTTCTCGGCGATAAAAT
                                            :ChrV 29648
```

junction sequence from *de novo* read alignment

D

```
                                                  ChrV 24590:
ATTGCGGAAGGAGCTTTGCTCTGGTTGAAATTATTATCATTATC:GCT:
ATTGCGGAAGGAGCTTTGCTCTGGTTGAAATTATTATCATTATC:GCT:TTGCATCTGATGCTGGCTCTACCGACTTCTCGGCGATAAAAT
                                            :GCT:TTGCATCTGATGCTGGCTCTACCGACTTCTCGGCGATAAAAT
                                            :ChrV 29648
```

junction sequence from PCR amplification and Sanger sequencing
