# Supplementary material for: DNA Repair Pathway Selection Caused by Defects in TEL1, SAE2, and De Novo Telomere Addition Generates Specific Chromosomal Rearrangement Signatures
Source: PLoS Genet. 2014 Apr 3;10(4):e1004277. doi: 10.1371/journal.pgen.1004277 (PMC3974649; doi:10.1371/journal.pgen.1004277)

Putnam et al. 2014. Figure S5 A.

Isolate 217

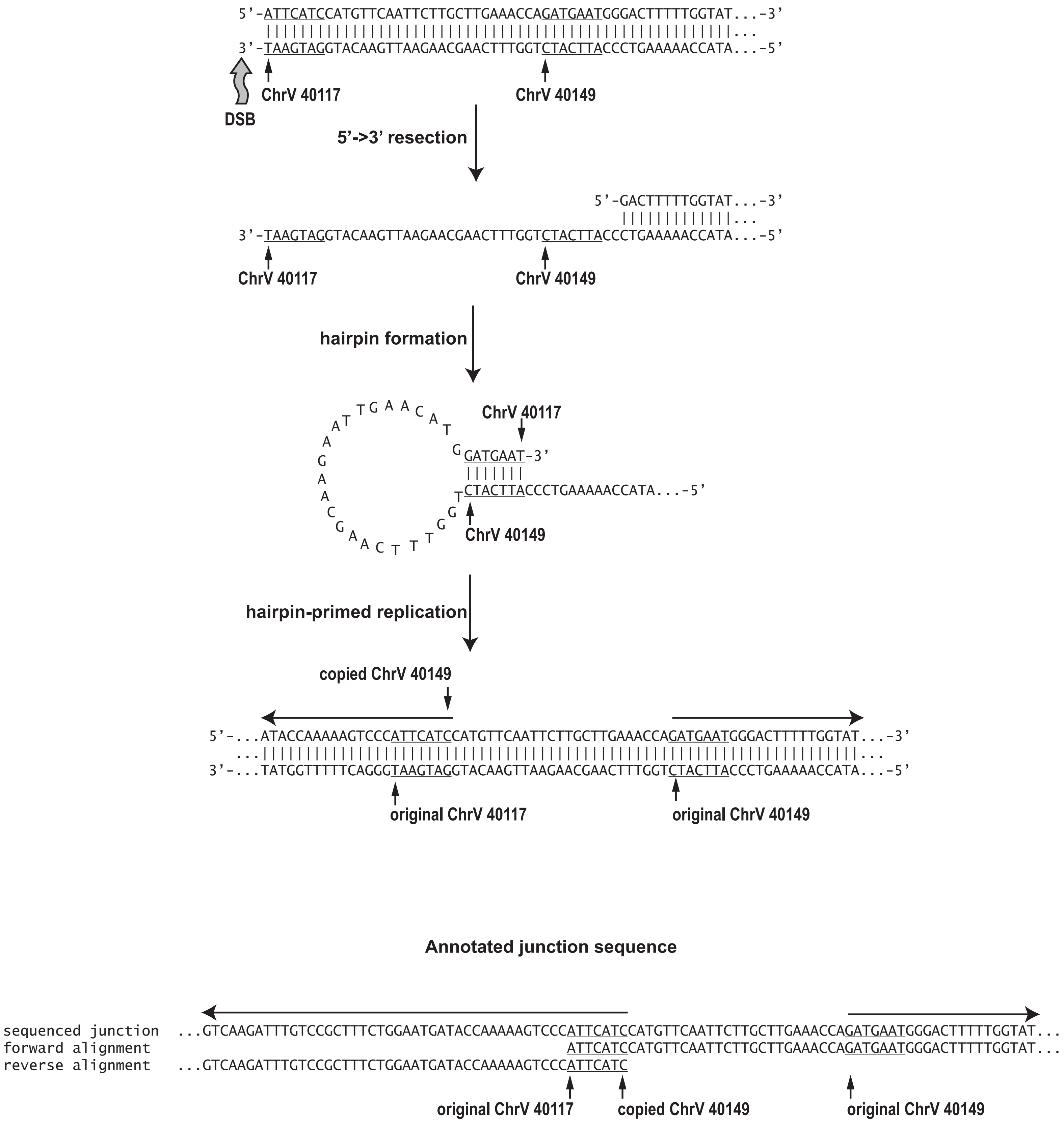

Putnam et al. 2014. Figure S5 B.

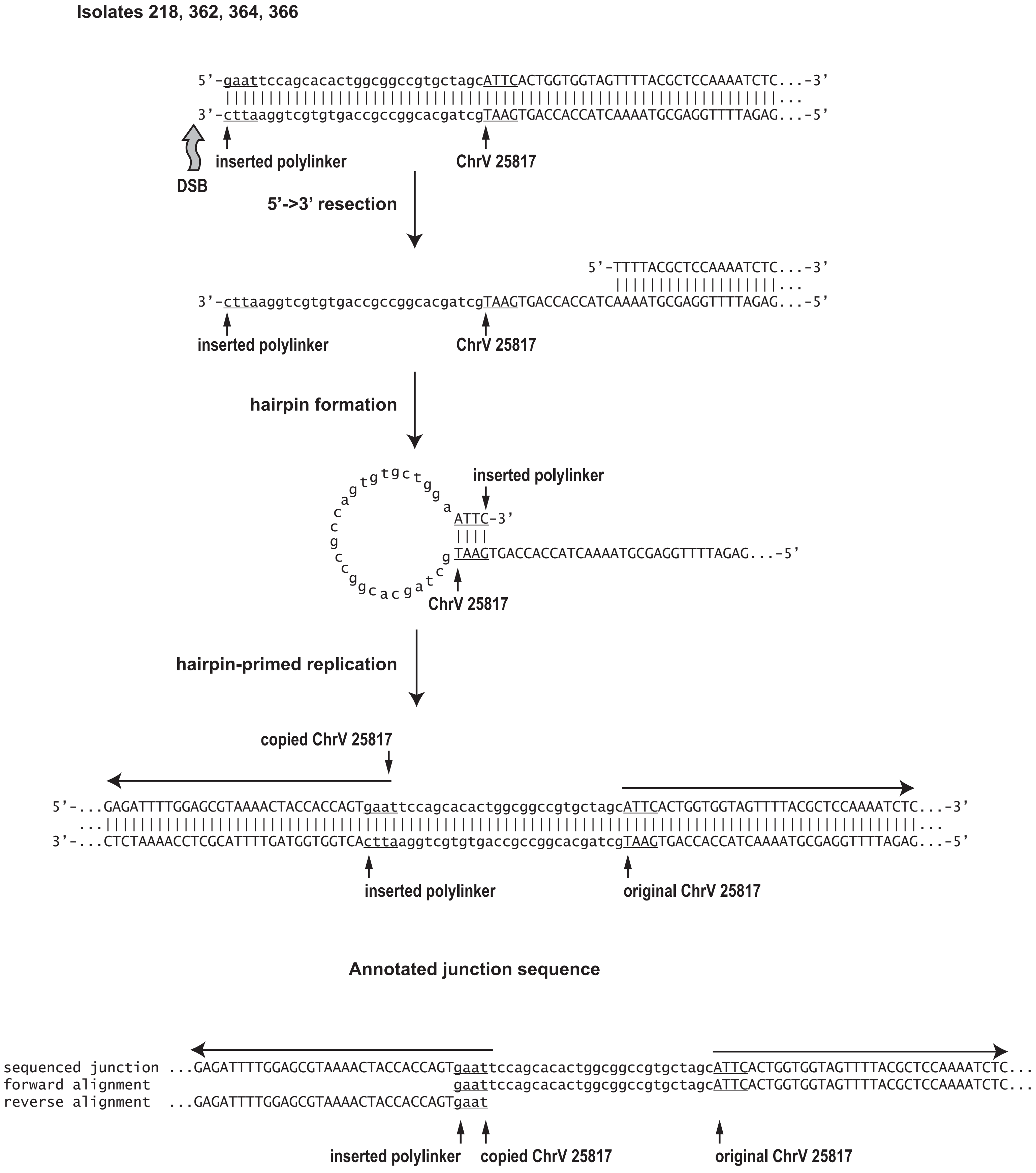

Putnam et al. 2014. Figure S5 C.

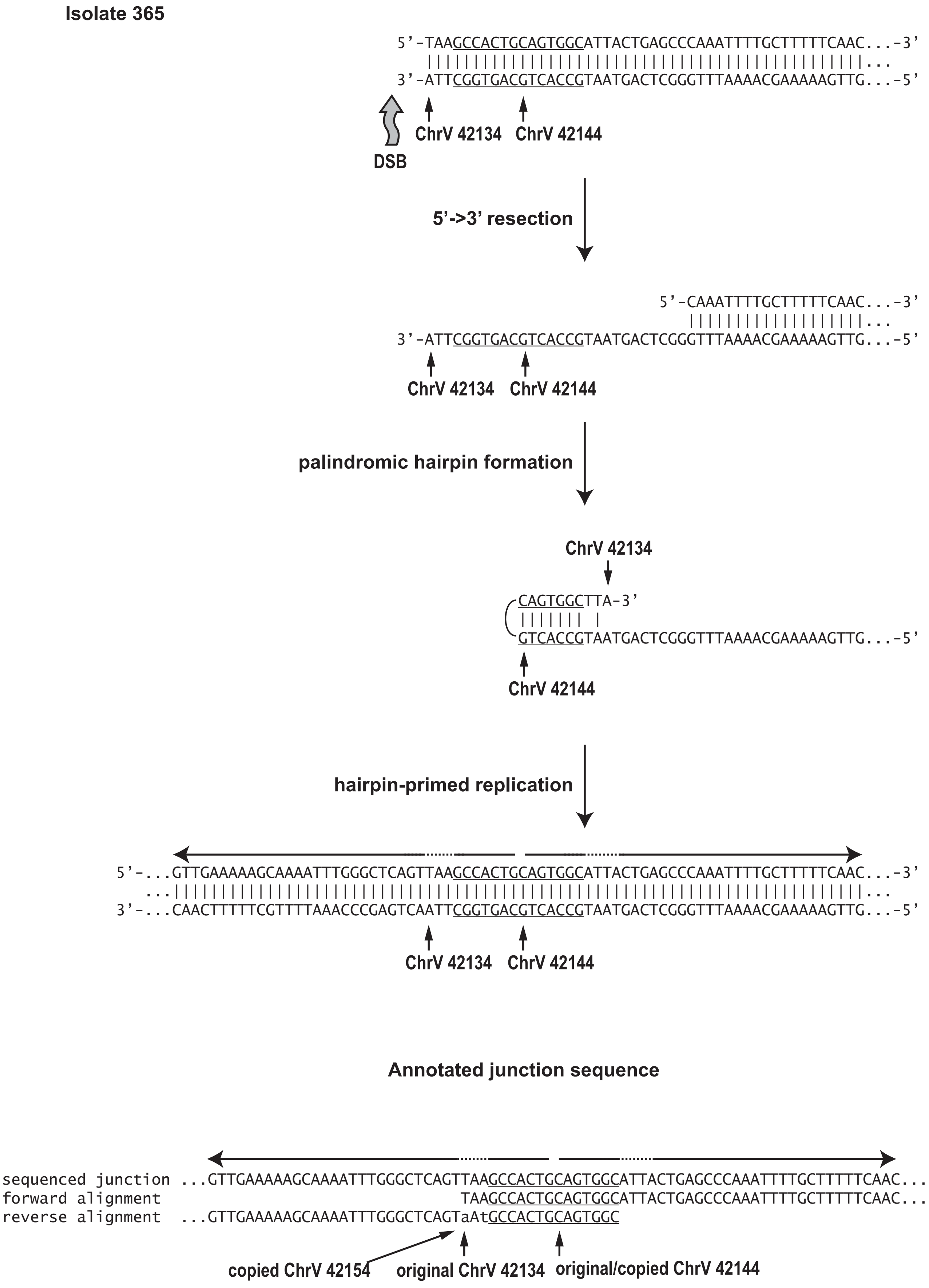

Putnam et al. 2014. Figure S5 D.

Isolate 2977 (in *hisG* insertion from *Samonella enterica* genome CP003416.1)

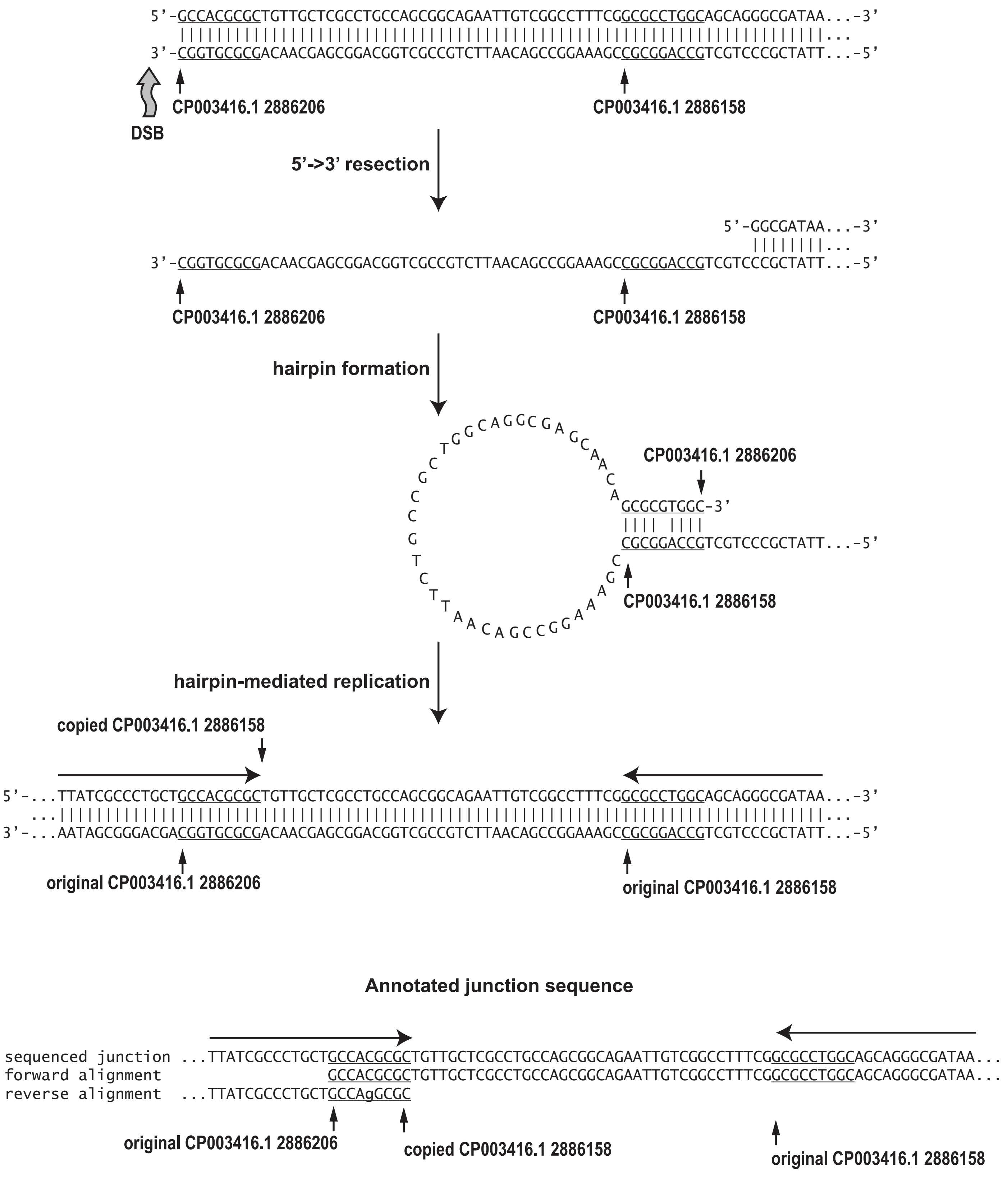

Putnam et al. 2014. Figure S5 E.

Isolate 3124 (in *CAN1* from the *CAN1/URA3* cassette with reversed orientation relative to reference sequence)

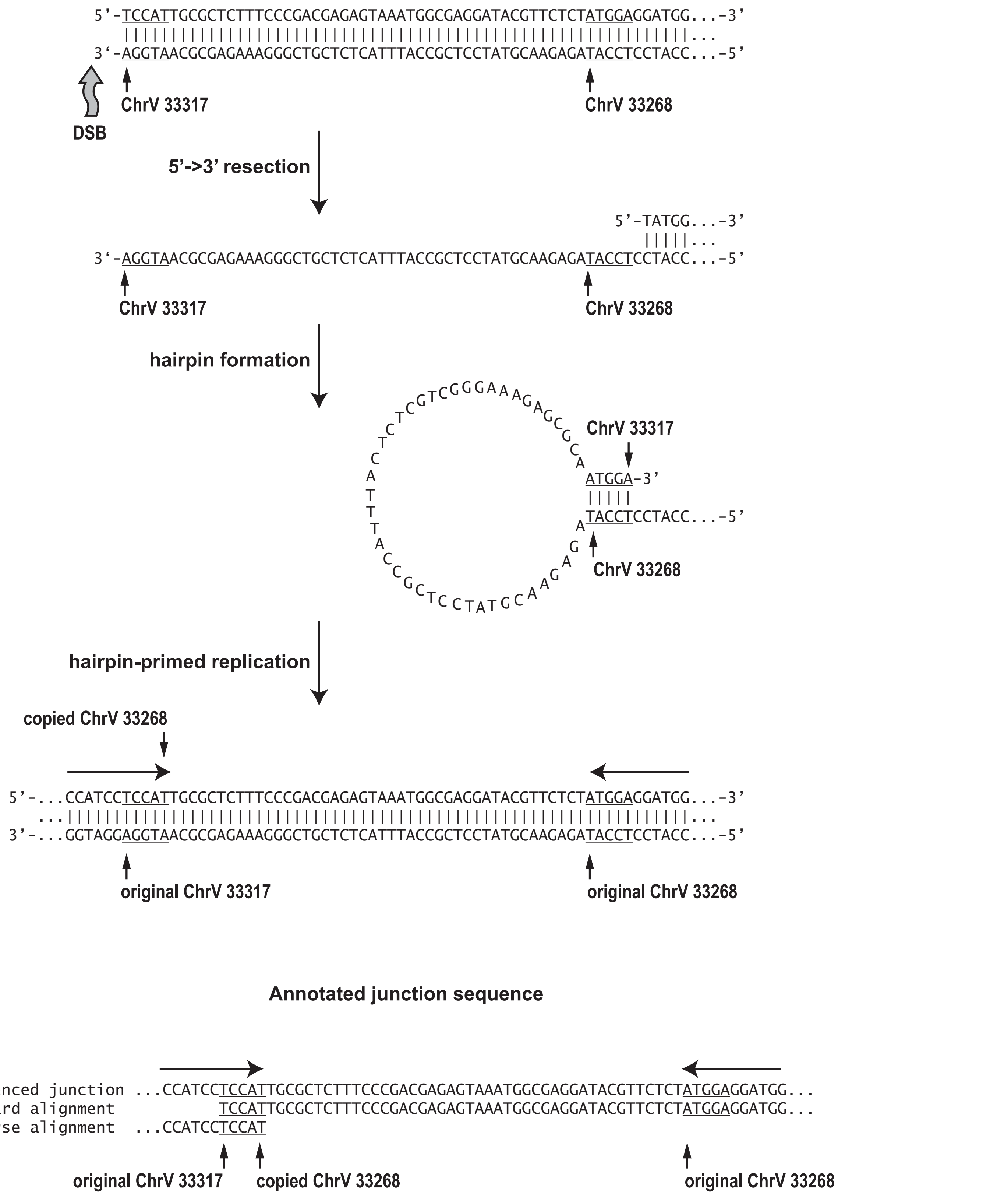

Putnam et al. 2014. Figure S5 F.

Isolates 3121, 3125 (in *hisG* insertion from *Samonella enterica* genome CP003416.1)

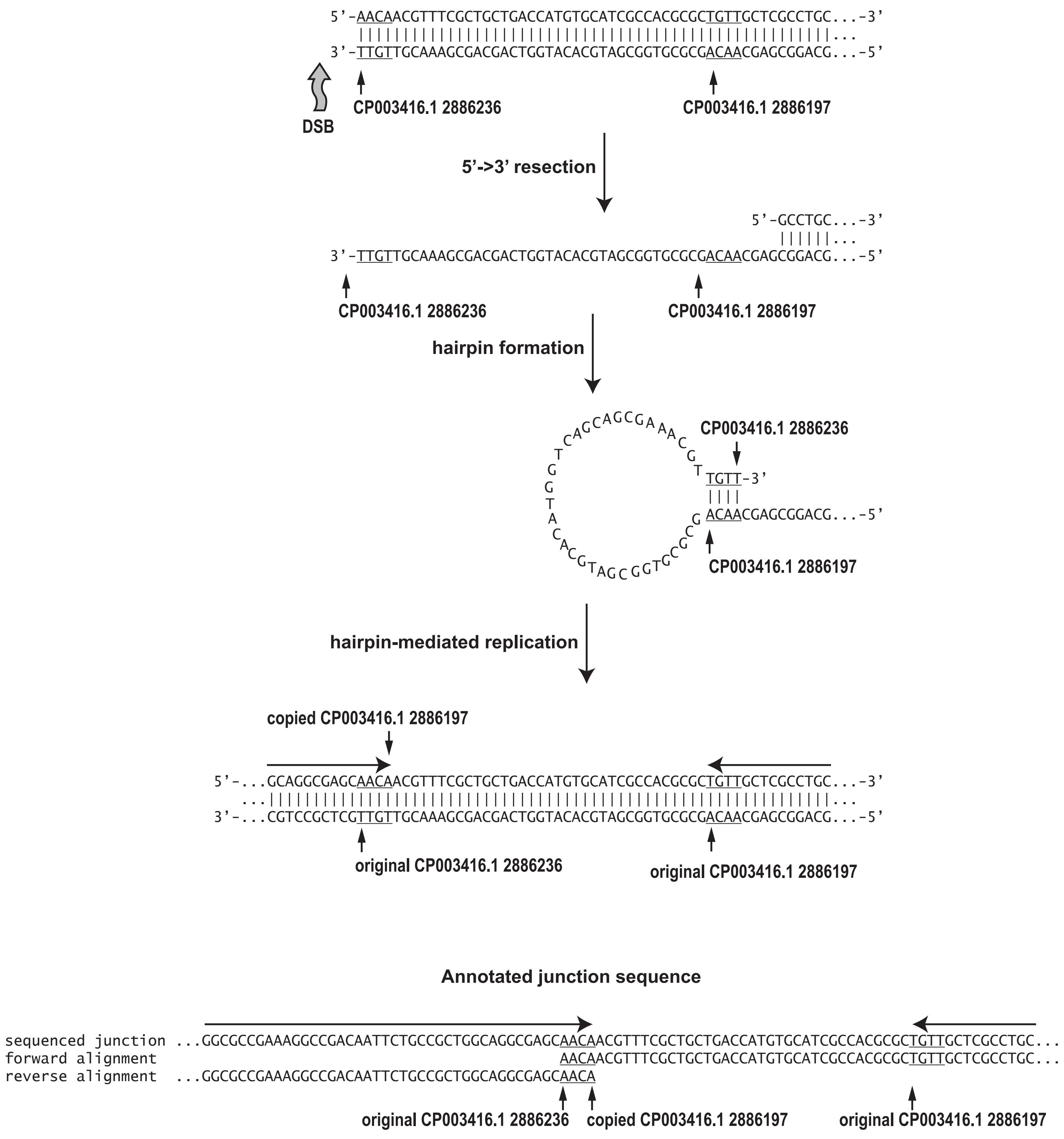

Putnam et al. 2014. Figure S5 G.

Isolate 3255 (from wild-type uGCR assay strain)

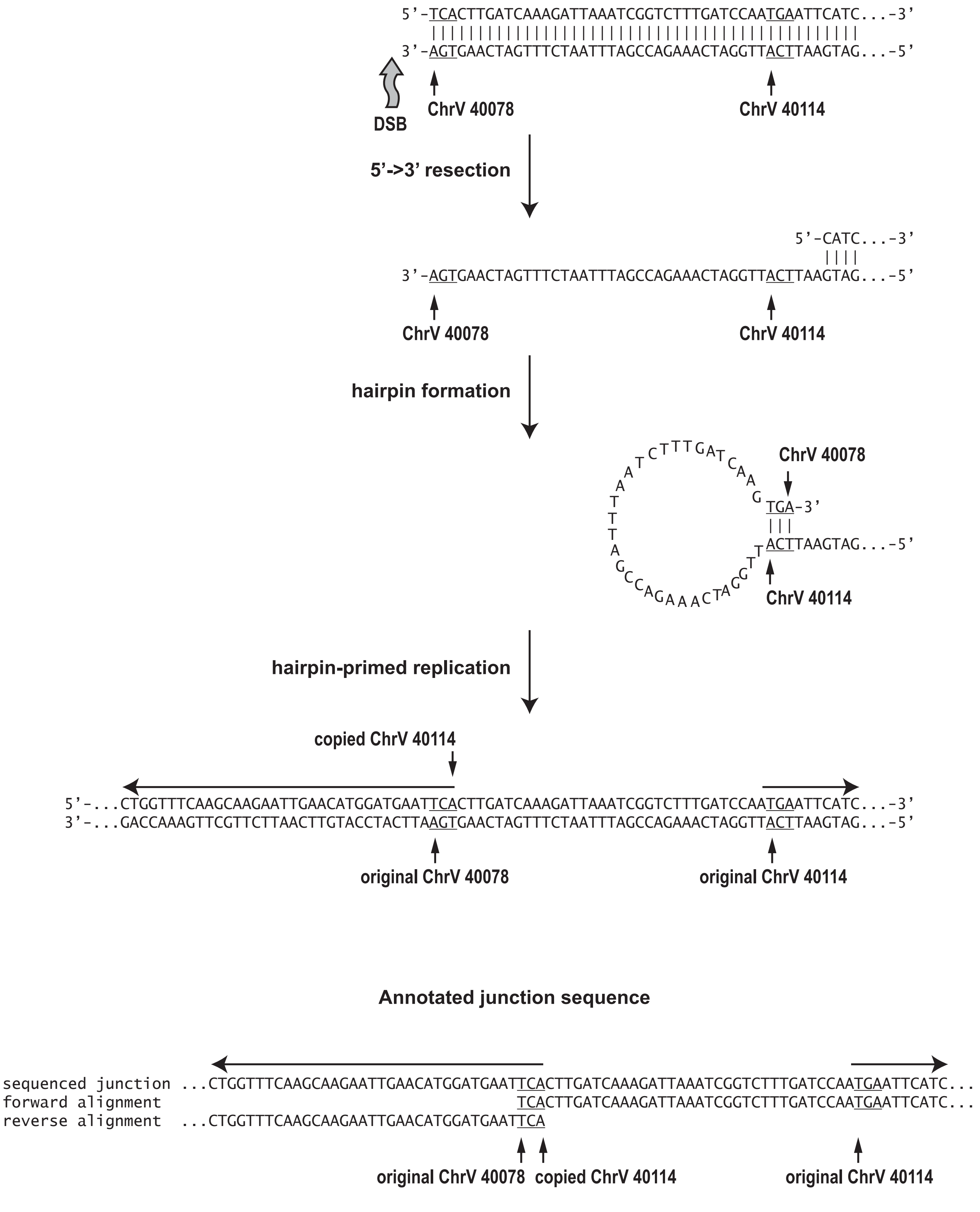

Supplement: Figure S5 — Formation and sequences of the inversion junctions in the sequenced inverted duplication GCRs. Predicted mechanism of hairpin formation and the sequence of the inversion junction for the inverted duplication class of GCRs. The arrow indicates the position of the double strand break (DSB) under the simplest scenario in which the 3′ end of the DSB is the 3′ end used to initiate the inversion; however, more complicated scenarios with more distal initiating DSBs have been observed [75]. 5′->3′ resection generates a single-stranded region that can mediate the formation of a single-stranded hairpin that can anneal and extend from the exposed single stranded region. Regions of homology involved in annealing are underlined. Duplicated palindromic sequence in the replicated product and the annotated junction sequence are displayed with arrows. Sequences derived from alignment of the junction sequencing reads are displayed at bottom. Panel A depicts the inversion junction for isolate 217; panel B depicts the inversion junction for isolate 218, 362, 364, and 366; panel C depicts the inversion junction for isolate 365; panel D depicts the inversion junction for isolate 2977; panel E depicts the inversion junction for isolate 3124; panel F depicts the inversion junction for isolates 3121 and 3125; and panel G depicts the inversion junction for isolate 3255. (PDF) [file pgen.1004277.s005.pdf]
