# Supplementary material for: DNA Repair Pathway Selection Caused by Defects in TEL1, SAE2, and De Novo Telomere Addition Generates Specific Chromosomal Rearrangement Signatures
Source: PLoS Genet. 2014 Apr 3;10(4):e1004277. doi: 10.1371/journal.pgen.1004277 (PMC3974649; doi:10.1371/journal.pgen.1004277)
Supplement: Figure S6 — Analysis of hph− GCR-containing isolates from the tel1Δ uGCR assay strain. (A) Southern blot using a MCM3 probe of a PFG of the wild-type strain (RDKY6677) and 13 hph− GCR isolates revealed that isolates 213, 2976, 3124, and 3125 had a rearranged chrV that was substantially larger than wild-type, whereas the other isolates had rearranged chrV that was similar to wild-type. (B) Sequences of some of the breakpoints from GCRs associated with a normal-sized chrV showed the GCRs involved translocations (isolate 213) or de novo telomere additions (isolates 3116, 3117, and 3120). For isolate 216, the junction sequence is displayed as in Figure S2 and the Ty elements in the reference genome that best matched the junction sequence were YJRWTy1-2 and YGRWTy1-1. (PDF) [file pgen.1004277.s006.pdf]

Putnam et al. 2014. Figure S6.

A

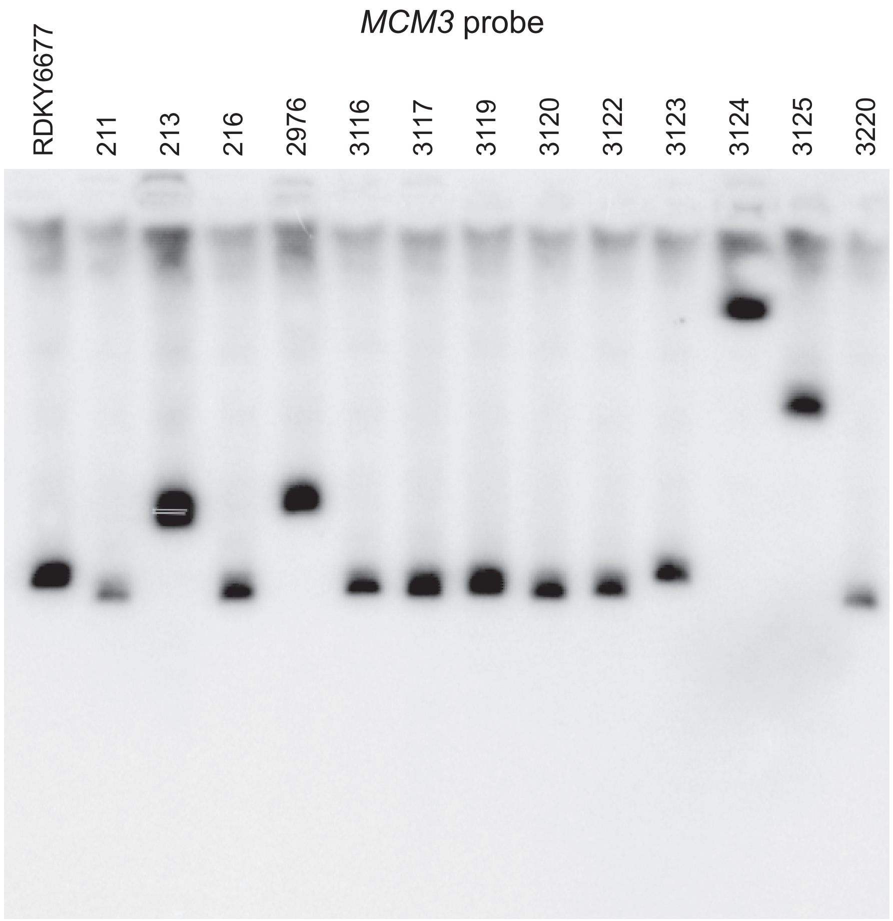

B

```
          ChrV 28264:
     GGTGTTGATGATAA:ATG:GTAAAGCTGGAATGAAT
216  GGTGTTGATGATAA:ATG:TGAATGTTGAGATAATT
                   :ATG:TGAATGTTGAGATAATT
                   :YJRWTy1-2(YJRWdelta13) or
                   :YGRWTy1-1(YGRWdelta14)

           ChrV 26402:
3116 TGTACGTCTGAATGTC:GG:TGTGGGTGTG

                  ChrV 40403:
3117 GACGCCACCGCCACTTTATC:GGGT:GGGTGTGGTGTGTGGGTGTGGTGTGTGGGT

                  ChrV 39764:
3120 TGAATCAAATGTTTTCGTTTTC:GTGTGTGG:GTGTGGTGTGTGT
```
