# Supplementary material for: DNA Repair Pathway Selection Caused by Defects in TEL1, SAE2, and De Novo Telomere Addition Generates Specific Chromosomal Rearrangement Signatures
Source: PLoS Genet. 2014 Apr 3;10(4):e1004277. doi: 10.1371/journal.pgen.1004277 (PMC3974649; doi:10.1371/journal.pgen.1004277)
Supplement: Table S1 — Statistics for Next Generation Sequencing results. (PDF) [file pgen.1004277.s010.pdf]

Putnam et al. 2014. Supplemental Table 1. Statistics for Next Generation Sequencing results.

Sample	No. Read Pairs*	No. Uniquely Mapping Read Pairs	Median Intra-Read Pair Distance (bp)
RDKY6761	6,790,544 (9,763,765)	5,202,261	348
isolate 213	1,620,637 (1,666,387)	1,126,340	488
isolate 217	16,453,691 (19,084,799)	13,377,250	324
isolate 218	6,530,448 (6,626,828)	4,438,139	525
isolate 362	56,076,913 (84,345,281)	35,187,865	544
isolate 364	11,908,333 (13,904,325)	9,478,272	346
isolate 365	16,614,757 (19,002,149)	13,559,892	342
isolate 366	10,194,633 (16,590,843)	7,539,510	337
isolate 2977	10,752,560 (16,356,597)	8,028,543	336
isolate 3118	11,333,616 (14,827,179)	8,920,560	349
isolate 3121	5,311,381 (5,364,880)	4,093,630	521
isolate 3124	7,549,579 (9,568,626)	3,835,490	483
isolate 3125	6,406,402 (7,047,941)	3,772,812	487
isolate 3178	5,902,900 (5,946,098)	4,607,977	522
isolate 3255	6,143,074 (6,199,015)	4,809,048	537

*Numbers in parentheses indicate the number of read pairs prior to culling PCR duplicates. All reads were 50 bases long.
