# Supplementary material for: DNA Repair Pathway Selection Caused by Defects in TEL1, SAE2, and De Novo Telomere Addition Generates Specific Chromosomal Rearrangement Signatures
Source: PLoS Genet. 2014 Apr 3;10(4):e1004277. doi: 10.1371/journal.pgen.1004277 (PMC3974649; doi:10.1371/journal.pgen.1004277)
Supplement: Table S2 — Number of junction-defining read pairs and junction-sequencing reads for isolates with sequenced genomes. (PDF) [file pgen.1004277.s011.pdf]

Putnam et al. 2014. Supplemental Table 2. Number of junction-defining read pairs and junction-sequencing reads for isolates with sequenced genomes.

Sample	Relevant Genotype	*hph* status	GCR-related junction*	GCR Description
RDKY6761	*tel1::HIS3*	+	n.a.	No GCR (starting strain)
isolate 213	*tel1::HIS3*	-	3/0	ChrVL Inversion (in *PRB1*)
isolate 217	*tel1::HIS3*	+	63/116	ChrVL Inversion (in *PRB1*)
isolate 218	*tel1::HIS3*	+	0/31	ChrVL Inversion (in interrupted *YEL068C*)
isolate 362	*tel1::HIS3*	+	58/287	ChrVL Inversion (in interrupted *YEL068C*)
isolate 364	*tel1::HIS3*	+	33/131	ChrVL Inversion (in interrupted *YEL068C*)
isolate 365	*tel1::HIS3*	+	35/93	ChrVL Inversion (in *SOM1-PRB1* intergenic region)
isolate 366	*tel1::HIS3*	+	32/101	ChrVL Inversion (in interrupted *YEL068C*)
isolate 2977	*tel1::HIS3*	+	29/42	ChrVL Inversion (in *can1::hisG*)
isolate 3118	*tel1::HIS3*	+	572/114	ChrVL Interstitial deletion (between *HXT13* and *AVT2*)
isolate 3121	*tel1::HIS3*	+	7/26	ChrVL Inversion (in *can1::hisG*)
isolate 3124	*tel1::HIS3*	-	10/25	ChrVL Inversion (in *CAN1*)
isolate 3125	*tel1::HIS3*	-	1/15	ChrVL Inversion (in *can1::hisG*)
isolate 3178	wild-type	+	36/39	ChrVL Inversion (in *PRB1*)
isolate 3255	wild-type	+	160/8	ChrVL Interstitial deletion (in *CAN1/URA3* cassette)

Sample	ChrV/*hph* telomeric junction	ChrV/*hph* centromeric junction	*URA3/YEL068C* junction	*URA3/CAN1* junction	*CAN1/YEL068C* junction
RDKY6761	341/125	309/82	369/184	349/174	353/149
isolate 213	0/0	0/0	0/0	0/0	0/0
isolate 217	720/184	644/206	656/373	0/0	0/0
isolate 218	99/24	213/42	107/41	0/0	0/0
isolate 362	1252/330	1546/262	1416/435	0/0	0/0
isolate 364	660/167	654/190	628/278	0/0	0/0
isolate 365	658/165	654/204	644/307	0/0	0/0
isolate 366	476/142	445/121	436/241	0/0	0/0
isolate 2977	545/151	504/136	529/230	0/0	0/0
isolate 3118	598/127	501/147	0/0	0/0	0/0
isolate 3121	174/43	197/53	162/43	0/0	0/0
isolate 3124	0/0	0/0	0/0	0/0	161/64
isolate 3125	0/0	0/0	0/0	0/0	0/0
isolate 3178	148/32	202/48	131/43	0/0	0/0
isolate 3255	187/42	217/45	213/40	0/0	175/60

Sample	*can1::hisG* telomeric junction	*can1::hisG* centromeric junction	*tel1::HIS3* centromeric junction	*tel1::HIS3* telomeric junction	*trp1Δ63* interstitial deletion
RDKY6761	236/168	304/143	348/201	230/157	35/13
isolate 213	0/0	0/0	18/11	14/13	12/7
isolate 217	0/0	0/0	623/436	394/306	120/36
isolate 218	508/312	272/96	85/36	53/36	80/32
isolate 362	2270/1250	2246/922	1273/322	745/529	1008/282
isolate 364	806/544	826/510	547/518	323/343	71/18
isolate 365	0/0	0/0	488/377	320/256	180/46
isolate 366	477/418	518/402	416/277	261/237	61/15
isolate 2977	0/0	562/408	515/285	290/217	49/5
isolate 3118	456/378	444/248	521/318	297/245	93/24
isolate 3121	0/0	303/130	120/42	107/58	95/36
isolate 3124	195/143	181/82	207/62	108/94	81/18
isolate 3125	0/0	219/98	159/59	81/61	88/28
isolate 3178	0/0	0/0	n.a.	n.a.	89/21
isolate 3255	194/110	163/82	n.a.	n.a.	169/37

Sample	circular 2-micron plasmid	circular mitochondrial genome	*ade2::hisG* centromeric junction	*ade2::hisG* telomeric junction	*MATa* centromeric junction*	*MATa* telomeric junction*
RDKY6761	132/21	46/28	23/6	29/13	15/9	120/0
isolate 213	759/179	0/0	19/6	22/7	0/0	11/0
isolate 217	635/131	184/99	132/30	119/34	17/49	153/0
isolate 218	5010/n.d.	0/0	78/39	85/34	0/0	55/0
isolate 362	n.d.	n.d.	1059/308	1026/250	737/5	9/0
isolate 364	320/79	53/34	70/17	57/12	13/19	170/0
isolate 365	706/156	143/80	206/39	186/56	13/21	142/0
isolate 366	248/62	94/49	41/22	47/17	18/46	114/0
isolate 2977	188/67	139/72	66/13	55/17	17/37	145/0
isolate 3118	248/45	154/68	70/29	71/29	11/26	173/0
isolate 3121	5362/n.d.	0/0	105/26	146/37	0/0	67/0
isolate 3124	7948/1210	0/0	101/66	88/46	0/0	83/0
isolate 3125	3119/574	10/15	107/29	107/28	1/0	66/0
isolate 3178	21/25	0/0	129/28	66/39	0/0	69/0
isolate 3255	5893/n.d.	0/0	143/23	140/27	0/0	104/0

Sample	*leu2Δ1* interstitial deletion	*his3Δ200* interstitial deletion
RDKY6761	281/68	30/9
isolate 213	29/4	23/10
isolate 217	600/155	109/0
isolate 218	120/24	91/38
isolate 362	861/234	1049/661
isolate 364	475/139	84/0
isolate 365	544/147	201/72
isolate 366	374/107	49/35
isolate 2977	399/123	55/22
isolate 3118	426/106	89/0
isolate 3121	133/20	137/33
isolate 3124	179/43	88/28
isolate 3125	125/35	87/40
isolate 3178	117/21	132/55
isolate 3255	190/36	167/43

*Results are reported as the number of junction-defining read pairs before the slash and the number of junction-sequencing reads after the slash. *MATa* associated rearrangements were identified as the reference genome is from a *MATα* strain. n.a., not applicable. n.d., not determined. Junctions with 0 junction-defining reads were found by alignment of non-mapping junction-sequencing reads but not through identification of junction-defining reads.
