# Supplementary material for: DNA Repair Pathway Selection Caused by Defects in TEL1, SAE2, and De Novo Telomere Addition Generates Specific Chromosomal Rearrangement Signatures
Source: PLoS Genet. 2014 Apr 3;10(4):e1004277. doi: 10.1371/journal.pgen.1004277 (PMC3974649; doi:10.1371/journal.pgen.1004277)
Supplement: Table S3 — S. cerevisiae strains. (PDF) [file pgen.1004277.s012.pdf]

Putnam et al. 2014. Supplementary Table 3. Yeast strains.

Name	*Genotype*	*Reference*
RDKY6677	*MATa leu2Δ1 trp1Δ63 his3Δ200 lys2ΔBgl hom3-10 ade2Δ1 ade8 ura3-52 iYEL072W::hph can1::hisG yel068c::CAN1/URA3*	Putnam et al. 2009
RDKY6678	*MATa leu2Δ1 trp1Δ63 his3Δ200 lys2ΔBgl hom3-10 ade2Δ1 ade8 ura3-52 iYEL072W::hph can1::hisG yel072w::CAN1/URA3*	Putnam et al. 2009
RDKY6764	RDKY6677 *chk1::HIS3*	Putnam et al. 2009
RDKY6727	RDKY6677 *ctf18::TRP1*	Putnam et al. 2009
RDKY6763	RDKY6677 *dun1::HIS3*	Putnam et al. 2009
RDKY8000	RDKY6677 *est1::G418*	This study
RDKY8002	RDKY6677 *est3::G418*	This study
RDKY6729	RDKY6677 *exo1::HIS3*	Putnam et al. 2009
RDKY8032	RDKY6677 *exo1::TRP1 sgs1::HIS3*	This study
RDKY8010	RDKY6677 *hta1-S129X.hisG hta2-S129X.hisG*	This study
RDKY8012	RDKY6677 *lig4::HIS3*	This study
RDKY8014	RDKY6677 *lig4::G418 tel1::HIS3*	This study
RDKY6760	RDKY6677 *mec1::HIS3 sml1::G418*	Putnam et al. 2009
RDKY6730	RDKY6677 *mrc1::TRP1*	Putnam et al. 2009
RDKY6779	RDKY6677 *mrc1::TRP1 tof1::HIS3*	Putnam et al. 2009
RDKY6766	RDKY6677 *mrc1-aq.TRP1*	Putnam et al. 2009
RDKY6848	RDKY6677 *mrc1-aq.TRP1 tof1::HIS3*	Putnam et al. 2009
RDKY6686	RDKY6677 *mre11::HIS3*	Putnam et al. 2009
RDKY8154	RDKY6677 *mre11::G418 tel1::HIS3*	This study
RDKY6894	RDKY6677 *pif1::HIS3*	Putnam et al. 2009
RDKY8008	RDKY6677 *pif1::HIS3 tel1::HIS3*	This study
RDKY6759	RDKY6677 *rad24::HIS3*	Putnam et al. 2009
RDKY6691	RDKY6677 *rad52::HIS3*	Putnam et al. 2009
RDKY8016	RDKY6677 *rad52::G418 tel1::HIS3*	This study
RDKY6762	RDKY6677 *rad53::HIS3 sml1::G418*	Putnam et al. 2009
RDKY6737	RDKY6677 *sae2::TRP1*	Putnam et al. 2009
RDKY8020	RDKY6677 *sae2::TRP1 exo1::HIS3*	This study
RDKY8022	RDKY6677 *sae2::TRP1 rad52::HIS3*	This study
RDKY8018	RDKY6677 *sae2::TRP1 tel1::HIS3*	This study
RDKY6687	RDKY6677 *sgs1::HIS3*	Putnam et al. 2009
RDKY8030	RDKY6677 *sir4::HIS3*	This study
RDKY6740	RDKY6677 *slx5::TRP1*	Putnam et al. 2009
RDKY6846	RDKY6677 *slx8::G418*	Putnam et al. 2009
RDKY6761	RDKY6677 *tel1::HIS3*	Putnam et al. 2009
RDKY6767	RDKY6677 *tof1::HIS3*	Putnam et al. 2009
RDKY8155	RDKY6677 *xrs2-11.HIS3*	This study
RDKY8004	RDKY6677 *yku70::HIS3*	This study
RDKY8006	RDKY6677 *yku80::HIS3*	This study
RDKY6773	RDKY6678 *chk1::HIS3*	Putnam et al. 2009
RDKY6744	RDKY6678 *ctf18::HIS3*	Putnam et al. 2009
RDKY6772	RDKY6678 *dun1::HIS3*	Putnam et al. 2009
RDKY8001	RDKY6678 *est1::G418*	This study
RDKY8003	RDKY6678 *est3::G418*	This study
RDKY6746	RDKY6678 *exo1::HIS3*	Putnam et al. 2009
RDKY8033	RDKY6678 *exo1::TRP1 sgs1::HIS3*	This study
RDKY8011	RDKY6678 *hta1-S129X.hisG hta2-S129X.hisG*	This study

RDKY8013	RDKY6678 *lig4::HIS3*	This study
RDKY8015	RDKY6678 *lig4::G418 tel1::HIS3*	This study
RDKY6769	RDKY6678 *mec1::HIS3 sml1::G418*	Putnam et al. 2009
RDKY6747	RDKY6678 *mrc1::TRP1*	Putnam et al. 2009
RDKY6780	RDKY6678 *mrc1::TRP1 tof1::HIS3*	Putnam et al. 2009
RDKY6775	RDKY6678 *mrc1-aq.TRP1*	Putnam et al. 2009
RDKY6849	RDKY6678 *mrc1-aq.TRP1 tof1::HIS3*	Putnam et al. 2009
RDKY6689	RDKY6678 *mre11::HIS3*	Putnam et al. 2009
RDKY8155	RDKY6678 *mre11::G418 tel1::HIS3*	This study
RDKY6936	RDKY6678 *pif1::HIS3*	Putnam et al. 2009
RDKY8009	RDKY6678 *pif1::HIS3 tel1::HIS3*	This study
RDKY6768	RDKY6678 *rad24::HIS3*	Putnam et al. 2009
RDKY6708	RDKY6678 *rad52::HIS3*	Putnam et al. 2009
RDKY8017	RDKY6678 *rad52::G418 tel1::HIS3*	This study
RDKY6771	RDKY6678 *rad53::HIS3 sml1::G418*	Putnam et al. 2009
RDKY6754	RDKY6678 *sae2::TRP1*	Putnam et al. 2009
RDKY8021	RDKY6678 *sae2::TRP1 exo1::HIS3*	This study
RDKY8023	RDKY6678 *sae2::TRP1 rad52::HIS3*	This study
RDKY8019	RDKY6678 *sae2::TRP1 tel1::HIS3*	This study
RDKY6690	RDKY6678 *sgs1::HIS3*	Putnam et al. 2009
RDKY8031	RDKY6678 *sir4::HIS3*	This study
RDKY6757	RDKY6678 *slx5::TRP1*	Putnam et al. 2009
RDKY6847	RDKY6678 *slx8::G418*	Putnam et al. 2009
RDKY6770	RDKY6678 *tel1::HIS3*	Putnam et al. 2009
RDKY6776	RDKY6678 *tof1::HIS3*	Putnam et al. 2009
RDKY8157	RDKY6678 *xrs2-11.HIS3*	This study
RDKY8005	RDKY6678 *yku70::HIS3*	This study
RDKY8007	RDKY6678 *yku80::HIS3*	This study
