# Supplementary material for: DNA Repair Pathway Selection Caused by Defects in TEL1, SAE2, and De Novo Telomere Addition Generates Specific Chromosomal Rearrangement Signatures
Source: PLoS Genet. 2014 Apr 3;10(4):e1004277. doi: 10.1371/journal.pgen.1004277 (PMC3974649; doi:10.1371/journal.pgen.1004277)
Supplement: Table S4 — MLPA primers. (PDF) [file pgen.1004277.s013.pdf]

Putnam et al. 2014. Supplemental Table 4. MLPA primers.

Gene	Primer sequences*	Location**	Product Size (bp)
ORC3	gggttccctaagggttggaAAGATGAACGTC AGCGAGTT	ChrXII 141097-141116	78
	P- TGCTGACGCCCAAAGGtctagattggatctt gctggcac	ChrXII 141117-141132	
TFC8	gggttccctaagggttggaTTTCTCGAGGAA ACGTTCAATTT	ChrXVI 542345-542323	84
	P- AGAAAGCTTCACGGCTGACtctagattggat cttgctggcac	ChrXVI 542322-542304	
DED81	gggttccctaagggttggaTACCTGGTTGAA CGAACACGACATCA	ChrVIII 142350-142325	90
	P- AGAACGAAGAGGGCGAAGACTTtctagattg gatcttgctggcac	ChrVIII 142324-142303	
PCM1	gggttccctaagggttggaGCCAATGGTATC GGTGGTCCACAGTTGAA	ChrV 43936-43964<br>ChrV-u 48729-48757	96
	P- AAAACTACTGGCCTCCGAAGATTGGtctaga ttggatcttgctggcac	ChrV 43965-43989<br>ChrV-u 48758-48782	
VMA8	gggttccctaagggttggaGATGCTAAGCAA AAGATGGGGAGAGTTATGCA	ChrV 58531-58562<br>ChrV-u 63324-63355	102
	P- AACTGCTGCCTTTTCCTTGGCCGAAGTTtct agattggatcttgctggcac	ChrV 58563-58590<br>ChrV-u 63356-63383	
BUD16	gggttccctaagggttggaGTACATGGATAT GTGGGAAATAAGGCTGCAACGTT	ChrV 97763-97729<br>ChrV-u 102556-102522	108
	P- TCCCTTACAGTGTCTAGGCTGGGATGTGGAT tctagattggatcttgctggcac	ChrV 97698-97728<br>ChrV-u 102521-102491	
GEA2	gggttccctaagggttggaGAGGAAGCTATT AGCGAGGACGATGGCATTGAAGAAGA	ChrV 112258-112295<br>ChrV-u 117051-117088	114
	P- GCATATTCATTCAGAGAAGAGCACAAATGGC GCCtctagattggatcttgctggcac	ChrV 112296-112329<br>ChrV-u 117089-117122	
EAF5	gggttccctaagggttggaCTGCAGCTGATT CTTCAAAAGAGCATAACGGACGGTGTAAT	ChrV 122173-122213<br>ChrV-u 132890-132930	120
	P- GCGAAAGGTCATTGGTACGGACGACTGGAAA TTGGCTtctagattggatcttgctggcac	ChrV 122214-122250<br>ChrV-u 132931-132967	
YEA6	gggttccctaagggttggaCTAAATAGGCTG AAAAAGAATGCTGACCCTAGAGTTGCTGCAA T	ChrV 144411-144454<br>ChrV-u 155127-155170	126
	P- TTCAGGTGCTTTATCTGGTGCACTATCCGCA ATGCTGGTCtctagattggatcttgctggca c	ChrV 144455-144494<br>ChrV-u 155171-155210	
IRC22	gggttccctaagggttggaCCTATTCGAAAT TGTTAGCCCACCTATTTCCTTTTTCAACCCA CAAT	ChrV 150496-1505450<br>ChrV-u 161212-161166	132
	P-	ChrV 1505449-150407	

	TTTTATCAGTTCAGGTCATCTTTCTAGCCATCATCGGAGGTGTtctagattggatcttgctggcac	ChrV-u 161165-161123	
MNN1	gggttccctaagggttggaCAAATATGAATACGATCCATCAAAAACCTTCTGGGCCAATTGGAGGGATA	ChrV 154401-154450<br>ChrV-u 165117-165166	138
	P-TGAGCGCCAAAGTAGCCGGTCGTGGTATTGTATTGAGTTTAGGTTCtctagattggatcttgctggcac	ChrV 154451-154496<br>ChrV-u 165167-165212	

*Uppercase bases correspond to homologies to the yeast chromosome; lowercase bases are common amplification sequences for the MLPA procedure. “P-” indicates 5’ phosphorylation.
**ChrV corresponds to the coordinates in ChrV in the S288C strain, whereas ChrV-u corresponds to the coordinates for the ChrV modified for the uGCR assay (RDKY6677).
